# Predictors of Professional Responses in Nonprofit Mental Health Forums: Interpretable Machine Learning Analysis

**DOI:** 10.2196/74359

**Published:** 2026-01-05

**Authors:** Shuang Geng, Yanghui Li, Jie Wang, Peixuan Chen, Xusheng Wu, Zhiqun Zhang

**Affiliations:** 1 College of Management Shenzhen University Shenzhen, Guangdong China; 2 College of Artificial Intelligence Shenzhen University Shenzhen, Guangdong China; 3 School of Life Sciences Central South University Changsha, Hunan China; 4 Shenzhen Health Development Research and Data Management Center Shenzhen, Guangdong China; 5 Shenzhen Traditional Chinese Medicine Hospital Shenzhen, Guangdong China

**Keywords:** influencing factors, online mental health community, predictive analysis, response length, response quantity, sentiment analysis, theme analysis

## Abstract

**Background:**

Online mental health communities increase access and equity for patients seeking psychological support. User demand and professional contributions are key to their sustainability. While previous research has examined factors influencing physicians’ participation in online consultation platforms, limited attention has been given to how post characteristics affect the quantity and length of professional responses in nonprofit mental health communities.

**Objective:**

This study aims to examine how textual (ie, topic, sentiment, title length, and content length) and contextual (ie, page views and posting time) characteristics of inquiries in nonprofit mental health forums influence the quantity and length of responses from mental health professionals, providing insights for enhancing community interactions.

**Methods:**

We collected 18,572 question-and-answer records from a Chinese online mental health platform (August 2024-July 2025). Topic features were extracted using BERTopic, and sentiment features were obtained through a distilled Bidirectional Encoder Representations from Transformers–based sentiment classification model. Additional features were derived from post metadata. We compared 5 machine learning models and identified Light Gradient Boosting Machine as the best performer. We then applied Shapley Additive Explanations (SHAP) analysis to it to evaluate the feature contributions to the prediction of response quantity and length.

**Results:**

In virtual mental health communities, user inquiries fall into 7 topic categories: work, love, depression, boyfriends or girlfriends, school, marriage, and family. Depression-related topics negatively predict response quantity, whereas interpersonal, school, marriage, or family topics are positively correlated. SHAP analysis revealed that page views (SHAP value=0.187) and title length (SHAP value=0.073) are key factors in predicting response quantity, and content length (SHAP value=0.274), sentiment category (SHAP value=0.054), and title length (SHAP value=0.053) are key factors in predicting response length. Posts exhibiting negative emotions are positively related to both the predicted quantity and length of responses, and this effect becomes more pronounced as the degree of emotional intensity increases. Titles with 15-20 characters and content with more than 60 characters are positively correlated with responses, whereas titles with fewer than 7 characters have negative effects. Higher view counts and weekday posts also increase response likelihood.

**Conclusions:**

This study provides insights into how textual and contextual features of patient posts influence professional responses in nonprofit mental health forums. It enhances understanding of voluntary knowledge contribution behaviors in online mental health communities and offers practical guidance for optimizing platform functional design and user posting strategies. Future researchers are encouraged to address the limitations of this study, which focuses solely on response quantity and length, and to explore details of professional responses, such as by developing a comprehensive measure of response quality.

## Introduction

### Background

Mental health disorders are critical global health concerns that pose major challenges to both individual well-being and health care systems [1-4]. According to the World Health Organization, approximately 1 billion individuals (more than 12.5% of adults and adolescents) are affected by mental health disorders, which account for approximately 5% of disability-adjusted life years [5]. Individuals living with mental health conditions experience a high burden of illness and face an increased risk of mortality [6]. Therefore, strengthening mental health services has emerged as a notable research concern [7].

In recent years, an increasing number of online mental health communities (OMHCs) have been established, allowing mental health professionals from offline hospitals to offer services on these platforms. These online mental health platforms connect mental health service providers with patient users seeking psychological support and are gaining popularity [8,9]. The benefits of OMHCs are manyfold. For example, OMHCs effectively mitigate social prejudice and stigma associated with mental health disorders. Many individuals delay or avoid seeking professional help because of concerns about being labeled. The anonymity setting of these platforms alleviates such concerns and encourages people with mental health challenges to actively seek psychological support [10,11]. Moreover, OMHCs help address the time and geographical limitations of offline clinical services. This also addresses the uneven distribution of mental health resources and enhances the accessibility and equity of mental health services [12]. Given its importance, this study focuses on OMHCs to better understand the contributing behavior of health professionals on these platforms.

Prior studies on online mental health can be classified into 3 main categories: studies that focus on patient users (eg, user-generated content, user engagement patterns) [13-17], studies that focus on health professionals (eg, contribution behaviors) [18,19], and studies that focus on communities (eg, community quality and value cocreation) [20-22]. Existing studies on the contribution behavior of health professionals suggest that their active participation within the community determines the effectiveness of counseling services [18]. For OMHC managers, understanding the factors that influence the contribution behaviors of health professionals is crucial for improving both the efficiency and quality of community responses, promoting sustained user participation, and enhancing user retention and engagement [23]. From the perspective of patient users, when they seek medical advice and feelings of expression in OMHCs, their posts signal their need for informational or emotional support from professionals. Responses from professional community members may make them feel valued and accepted and enhance their sense of belonging to the community. Moreover, the information shared by professionals benefits not only help-seekers but also lurkers and other community members [24]. Together, the engagement of both patients and professionals helps cultivate a sustainable ecosystem for health knowledge exchange [25]. Therefore, it is imperative to focus on mental health professionals and examine the factors influencing their knowledge contributions in OMHCs.

Previous studies have provided insights into the factors influencing health professionals’ knowledge contribution behaviors [20,26-33]. These factors include content-related factors, such as question type, information quality, and readability [26-28]; community-related factors, such as reward mechanisms [26]; and health professional–related factors, such as social relationships, self-efficacy, and reputation needs [30-33]. For example, Srivastava et al [27] analyzed the intent, criticism, readability, and emotion of user posts on Reddit, and found the prominence of “self-criticism” as the most prevalent form of criticism expressed by help-seekers. They also found that individuals who explicitly express their need for help are more likely to receive assistance. These studies explore these factors primarily through the lens of signal theory, motivational theory, self-determination theory, and social exchange theory. For instance, Chen et al [28] reported that emotional and informational language signals increase the likelihood of professionals providing informational and emotional support. Drawing on social exchange theory, Wang et al [29] reported that material and psychological rewards significantly increase the online contributions of health professionals. Additionally, several studies have explored the impact of the intrinsic and extrinsic motivations of professionals’ contribution behaviors. Imlawi and Gregg [32] noted that factors such as helping motivator, reputation motivator, and moral obligation motivator influence professionals’ contribution continuance intentions. Maheshwari et al [33] found that self-efficacy and reciprocity positively influence the attitude toward knowledge sharing; however, the rewards’ moderating effect is not significant.

While there is a wide range of factors that influence professionals’ knowledge contribution, post characteristics serve as the most direct medium for patient users to share stories and engage with the community, and they are closely associated with social interaction outcomes [27]. The informational cues (eg, topic) and emotional cues (eg, sentiment) in a post are crucial signals for consultants to understand the patient users’ problem and to decide whether to respond. For example, posts with clear problem descriptions may reduce the cognitive effort required by consultants, which is especially important when the professionals are volunteers with limited time. Research in computer-mediated communication suggests that emotional cues are important drivers of social support [34]. Beyond content, posting time is a critical factor that determines a post’s visibility. For example, a post published late at night may be quickly buried under a flood of newer posts, making it receive fewer replies. Investigating the influences of these post features can provide actionable insights beyond those gained from focusing on user or professional factors. Demographic or professional attributes of users and professionals are often static and difficult to change within a platform’s context. In contrast, content features are dynamic. Community managers can develop posting guidelines and platform functionalities to help patients craft more effective queries. Therefore, this study aims to address this research gap by investigating diverse post features and providing practical implications that empower users to effectively seek and obtain professional responses. This study conducts a microlevel analysis of post features, which complements the user-level and community-level research in the OMHC landscape. Our work offers granular and actionable explanations for knowledge contribution in OMHCs.

Previous online health community research has usually adopted structural equation modeling, multiple linear regression, and fixed effect modeling to analyze the relationships between focal factors [29,35,36]. The rise of artificial intelligence and machine learning has provided powerful tools for modeling, partitioning, and interpreting the complex relationships between factors. A few recent mental health studies have used machine learning algorithms, such as logistic regression, decision trees, and ensemble models [37-39]. Light Gradient Boosting Machine (LightGBM), an ensemble learning algorithm based on gradient boosting decision trees, is widely used for classification, regression, and ranking tasks because of its efficiency and outstanding performance, particularly in structured and tabular data problems [38]. However, LightGBM, like other ensemble models (eg, Extreme Gradient Boosting [XGBoost]), is often regarded as a “black-box” model and lacks sufficient transparency and interpretability. Explainable machine learning techniques such as Shapley Additive Explanations (SHAP) [39] can visualize the importance of factors in driving model decisions, enabling stakeholders to understand the logic behind the model’s outputs and make informed decisions. Therefore, this study combines these 2 techniques to detect important post features that affect the quantity and length of professional responses in OMHCs, aiming to improve community interaction quality and enhance the effectiveness of mental health services.

### Objective

The research question for this study is as follows: “In nonprofit OMHCs, how do patient posts’ textual features (eg, topic, sentiment, title length, and content length) and contextual features (eg, page view and posting time) influence the response quantity and length of health professionals?” To answer this question, this paper constructs an interpretable machine learning model for analysis. This study aims to deepen the understanding of knowledge contribution in OMHCs and to inform management strategies for building more supportive and efficient mental health communities.

## Methods

### Overview

**Figure 1:**
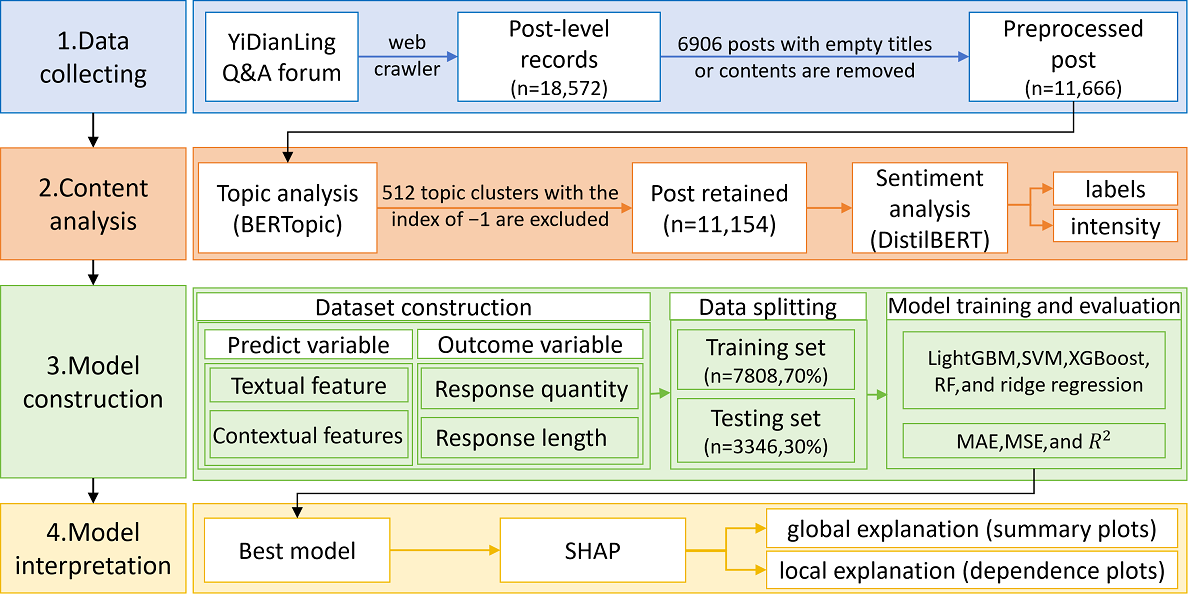
Study flowchart. Panels 1-2 depict the data collection and processing workflow, and panels 3-4 illustrate the development of the interpretable machine learning model. LightGBM: Light Gradient Boosting Machine; MAE: mean absolute error; MSE: mean squared error; Q&A: question-and-answer; RF: random forest; SHAP: Shapley Additive Explanations; SVM: Support Vector Machine; XGBoost: Extreme Gradient Boosting.

The research design for this study is shown in Figure 1. This framework consists of 4 main phases: data collection, thematic and sentiment analysis, predictive model construction, and predictive model interpretation. In the data collection process, we collected a total of 18,572 post-level records using a web crawler. Subsequently, we performed preliminary data cleaning to remove posts with empty titles or empty content. We then analyzed the post topics using BERTopic and removed noisy clusters. A total of 11,154 data entries were obtained after noise (topic cluster index=–1) removal. Sentiment analysis was conducted using distilled Bidirectional Encoder Representations from Transformers (DistilBERT) to determine both the category and intensity of sentiment for each post. During the modeling phase, we trained and compared 5 models (ie, LightGBM, Support Vector Machine [SVM], XGBoost, random forest [RF], ridge regression) to select the best one to develop the final model. These models were trained using textual and contextual features of the posts as predictive variables. The models were designed to predict 2 key outcome variables: response quantity and response length. Finally, we used the SHAP method to interpret the model. It provided global interpretations via summary plots to determine overall feature importance and local interpretations using dependence plots to elucidate how individual features influence the model’s predictions.

### Data Collection

We collected data from the YiDianLing platform [40]. YiDianLing is a leading nonprofit mental health service provider in China, which hosts approximately 50 million registered users and 60,000 professional psychological consultants. The large and diverse user base ensures the representativeness of the data. Moreover, the platform’s nationwide coverage provides broad geographic representation. YiDianLing provides a dedicated public question-and-answer forum to facilitate interaction between patient users and certified psychological consultants. In this forum, users can anonymously post their mental health concerns, and psychological consultants can provide free responses. Therefore, the forum generates rich data, including user posts, consultant responses, post view counts, and response volume.

**Figure 2:**
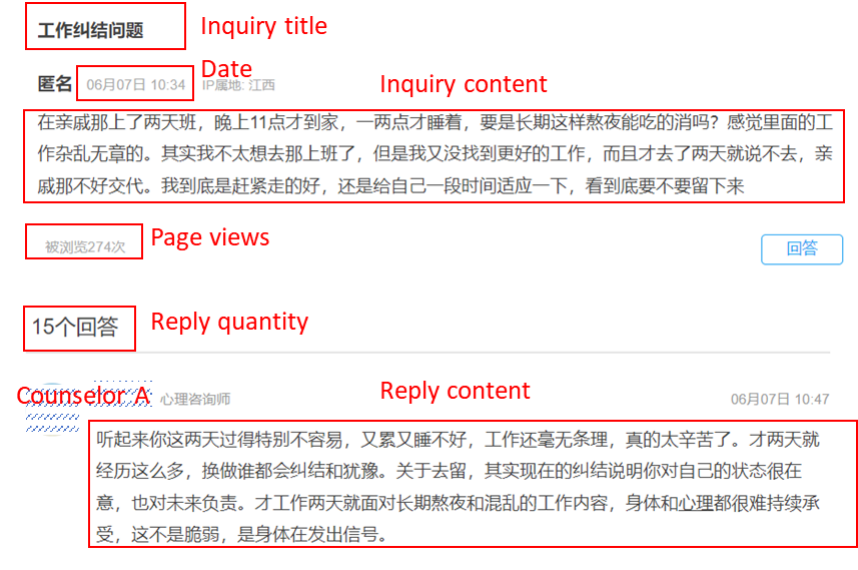
Example of extracted features from the post.

Data from the YiDianLing, which comprised 18,572 entries from August 2024 to July 2025, were collected using a crawler program. An example of the data source page is depicted in Figure 2. Each data sample includes information about the user’s post, such as the post title, post content, date of post, number of page views, number of responses, and number of responses provided by psychological consultants. The temporal evolution of the number of posts and the number of responses on the platform is presented in Multimedia Appendix 1. The volume of posts and responses exhibits a high degree of stability, with the number of replies consistently exceeding the number of posts.

### Content Analysis

#### Topic Analysis

BERTopic is a topic modeling technique based on Bidirectional Encoder Representations from Transformers (BERT), which is a pretrained language model based on the transformer architecture that reads text in both directions (left-to-right and right-to-left) to understand the full context [41]. This architecture is based on multilayer neural networks called encoders, and it uses a self-attention mechanism to capture word relationships and context. BERTopic can effectively add interoperability challenges between density-focused clustering and centroid-oriented methods. This study uses the BERTopic model for thematic analysis, as it has demonstrated advantages in various topic modeling benchmark tests [42].

BERTopic facilitates consistent topic identification by leveraging a category-specific version of the term frequency-inverse document frequency (TF-IDF). In this technique, all text in a cluster is considered one entity, and TF-IDF is applied to determine the relevance scores for words within that cluster. By extracting important words in each cluster, descriptions of topics are obtained. This method is known as class-based TF-IDF:

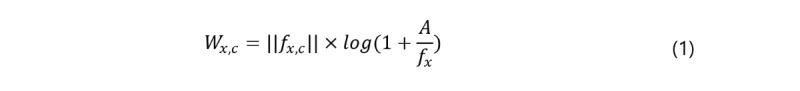



where *f_x,c_* represents the frequency of word *x* in cluster *c*, *f_x_* denotes the frequency of word *x* across all clusters, and *A* signifies the average number of words contained in each cluster.

#### Sentiment Analysis

In this study, we used the DistilBERT method to classify post sentiment into 5 categories: very negative, negative, neutral, positive, and very positive [43]. This model is a lightweight pretrained language model built on BERT through knowledge distillation techniques [44]. DistilBERT retains BERT’s performance in capturing the sentence context and reduces the number of parameters to achieve higher computational efficiency. The used DistilBERT model has been fine-tuned on a multilingual corpus, including Chinese, and has been successfully applied to a wide range of tasks, such as product review classification, social media sentiment analysis, and customer feedback analysis [45,46].

In the sentiment analysis, we first segmented each user post into a set of sentences and applied DistillBERT analysis separately. The predicted sentiment labels were then mapped to numerical scores, with “very negative” as –1, “negative” as –0.5, “neutral” as 0, “positive” as 0.5, and “very positive” as 1. The sentiment scores of the set of sentences were then summed to obtain an overall sentiment score for the post. On the basis of this overall sentiment score, we classified each post into one of three sentiment polarities: posts with sentiment scores less than 0 were classified as negative, posts with a score equal to 0 were classified as neutral, and posts with a score greater than 0 were classified as positive. The sentiment intensity of each post was measured as the logarithm of the absolute value of the overall sentiment score plus 1.

### Model Construction

LightGBM is an efficient ensemble method built on gradient boosting decision trees, which construct multiple classifiers and integrate their outputs to obtain the final prediction [47]. It is widely applied to classification, regression, and ranking tasks and can model structured and tabular data [38,48]. Unlike RFs and XGBoost, LightGBM adopts a histogram-based splitting technique that splits data and scans the statistics to determine the best split point, which enables less memory consumption and more efficient training [48].

We compared 5 popular machine learning models: LightGBM, SVM, XGBoost, RF, and ridge regression. We used a 70/30 train-test split, and the data are randomly divided 5 times to reduce the randomness introduced by data splits. The average performance of the 5 trained models is used for final model evaluation using mean absolute error, mean squared error, and R-squared. Based on these evaluations (Multimedia Appendix 2), LightGBM showed the best performance and was selected for subsequent regression prediction and interpretation.

During model training, hyperparameters were tuned on the basis of the official LightGBM documentation [49]. Specifically, the maximum number of leaves per weak learner was set to 40 to mitigate overfitting; the learning rate was set to 0.05 to accelerate convergence and improve prediction accuracy; and feature_fraction was set to 0.8, enabling the model to randomly select a subset of features when constructing each tree, thereby reducing training time. The detailed hyperparameter settings are provided in Multimedia Appendix 3.

### Model Interpretation

The black-box nature of traditional machine learning models, such as ensemble methods and neural networks, limits their clinical application in the mental health domain, as stakeholders require transparent and trustworthy decision-making processes [50]. SHAP is a model interpretation method based on cooperative game theory [51]; it provides a unified measure of feature importance by attributing the model’s prediction to the marginal contributions of each feature, known as SHAP values. It works as follows.

Consider the *i^th^* sample as *x_i_*, where *x_ij_* represents the *j^th^* feature of the *i^th^* sample, and *y_i_* denotes the model’s forecast for this sample. The baseline model prediction (often the predicted mean of all samples) is denoted *y_base_*. The SHAP value is then derived based on the following formula:

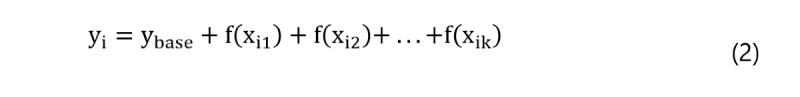



where *f*(*x_ij_*) denotes the SHAP value for *x_ij_*, indicating the influence of the *j^th^* feature of the *i^th^* sample on the ultimate prediction *y_i_*. A positive value suggests that the feature enhances the prediction, whereas a negative *f*(*x_ij_*) indicates a diminishing effect on the predicted outcome.

We conducted SHAP analysis and visualized the contribution of each feature to the model’s prediction using importance ranking summary plots. The dependence plots show the relationship between the changes in a feature’s value and its impact on the prediction. The quantified contributions of features, either positive or negative, enhance the transparency of the prediction model.

### Ethical Considerations

This study did not involve human participants. The data were publicly accessible information on the YiDianLing platform. All user posts in the question-and-answer forum were published anonymously by patient users, and no identifiable or reidentifiable personal information was collected or processed throughout the research. Therefore, there is no risk to individual privacy or foreseeable harm to users. We conducted our data collection in accordance with the platform’s data authorization agreement and ensured that all procedures fully complied with the relevant ethical standards. During the handling of the dataset, we also took steps to maintain data security. This research project received formal approval from the Institutional Review Board of Shenzhen University (approval number PN-202500199).

## Results

### Topic Analysis and Sentiment Analysis

**Figure 3:**
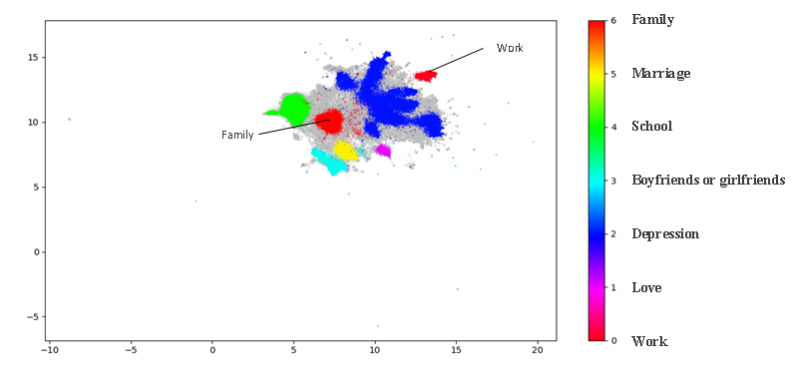
Topic clustering results.

The results of the topic modeling visualization revealed 8 themes of user posts, as shown in Figure 3. The gray areas in Figure 3 are clusters with a category index of –1 and are considered noise and were excluded from the analysis. Representative keywords for each topic are provided in Multimedia Appendix 4.

**Figure 4:**
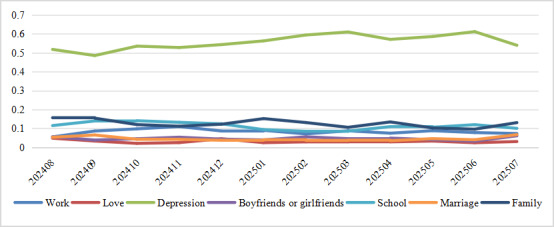
Topic frequency evolution.

On the basis of the results of thematic clustering, we visualize the frequency of different themes over the observation period in Figure 4. The results indicate that depression consistently remained the predominant theme. Its proportion significantly exceeds that of the other categories, followed by family, school, and work. The overall topic frequency demonstrated stability throughout the period.

**Figure 5:**
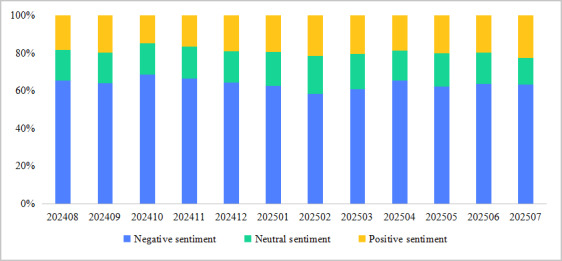
Emotional map for different dates.

The proportions of sentiment categories at different times are depicted in Figure 5, which shows that the proportion of posts with negative sentiment is greater than those with positive and neutral sentiment. The distribution of sentiment categories remains relatively stable over time, indicating that patient users primarily express negative emotions in the mental health community.

### Descriptive Statistics

A total of 11,154 data entries were obtained after topic clustering and noise removal and were used for analysis. The predictor variables included post theme, sentiment category, sentiment intensity, title length, content length, posting year, posting month, posting date, public holiday status, time of day, day of the week, and page view count. The outcome variables comprise the quantity and length of replies, and prediction models are constructed separately for each. We acknowledge that a full picture of community interaction also depends on the quality of responses, a multifaceted construct that encompasses aspects like relevance, empathy, and supportiveness. Capturing this richness quantitatively poses a distinct methodological challenge. We therefore view our work as a critical first step that sets the stage for, and thereby invites, subsequent research to delve into the nuanced quality of professional contributions. A description of the features is provided in Table 1. Descriptive statistics of these selected variables are presented in Table 2, excluding the temporal characteristics of the posts.

**Table 1:** Description of features.

Feature	Feature description	Variable type
Topic	The topic of the content; 0=work; 1=love; 2=depression; 3=boyfriends or girlfriends; 4=school; 5=marriage; 6=family	Categorical variable
Sentiment category	0=negative; 1=neutral; 2=positive	Categorical variable
Sentiment intensity	The logarithm of the absolute value of the sentiment score plus 1	Continuous variable
Page view	The logarithm of the number of page views posted on a given date	Continuous variable
Year	Year of the post	Categorical variable
Month	Month of the post	Categorical variable
Day	Day of the post	Categorical variable
Title length	The natural logarithm (base e) of the number of Chinese characters in the post title (note: raw character counts are used for result interpretation)	Continuous variable
Content length	The natural logarithm (base e) of the number of Chinese characters in the post content (note: raw character counts are used for result interpretation)	Continuous variable
Hour	0=00:00~00:59, 1=01:00~01:59, 2=02:00~02:59, 3=03:00~03:59, 4=04:00~04:59, 5=05:00~05:59, 6=06:00~06:59, 7=07:00~07:59, 8=08:00~08:59, 9=09:00~09:59, 10=10:00~10:59, 11=11:00~11:59, 12=12:00~12:59, 13=13:00~13:59, 14=14:00~14:59, 15=15:00~15:59, 16=16:00~16:59, 17=17:00~17:59, 18=18:00~18:59, 19=19:00~19:59, 20=20:00~20:59, 21=21:00~21:59, 22=22:00~22:59, 23=23:00~23:59	Categorical variable
Week	0=Monday; 1=Tuesday; 2=Wednesday; 3=Thursday, 4=Friday; 5=Saturday; 6=Sunday	Categorical variable
Holiday	0=no; 1=yes	Categorical variable
Reply quantity	The logarithm of the number of replies to the post	Continuous variable
Reply length	The logarithm of the average reply length of the post	Continuous variable

**Table 2:** Descriptive statistics.

Features		Value (n=11,154)
Page view, mean (SD)		5.09 (0.67)
**Sentiment category, n (%)**		
	Positive	2117 (19)
	Neutral	1904 (17.1)
	Negative	7133 (63.9)
Sentiment intensity, mean (SD)		0.69 (0.48)
**Topic, n (%)**		
	Work	917 (8.2)
	Love	355 (3.2)
	Depression	6139 (55)
	Boyfriends or girlfriends	519 (4.7)
	School	1269 (11.4)
	Marriage	501 (4.5)
	Family	1454 (13)
Title length, mean (SD)		2.92 (0.40)
Content length, mean (SD)		4.29 (1.30)
Reply quantity, mean (SD)		0.22 (0.66)
Reply length, mean (SD)		5.25 (0.83)

### Comparisons of Model Performance

We evaluated 5 machine learning models: LightGBM, SVM, XGBoost, RF, and ridge regression for predicting response quantity and response length. Tables 3 and 4 present the performance of these models. Results show that LightGBM achieved the lowest mean absolute error (mean 0.2859, SD 0.0072) and mean squared error (mean 0.3100, SD 0.0142), along with the highest *R*^2^ (mean 0.2754, SD 0.0323), statistically outperforming SVM, XGBoost, RF, and ridge regression. Similarly, for response length prediction (Table 4), LightGBM demonstrated superior overall performance. These metrics provide a comprehensive assessment of each model’s capabilities. Additionally, calibration curves for the LightGBM model in both prediction tasks are provided in Multimedia Appendix 2, both of which indicate a good model fit.

**Table 3:** Response quantity prediction performance of the compared models.

Model	MAE^a^, mean (SD)	MSE^b^, mean (SD)	*R*^2^, mean (SD)
LightGBM^c^	0.2859 (0.0072)	0.3100 (0.0142)	0.2754 (0.0323)
SVM^d^	0.3101 (0.0161)	0.3931 (0.0391)	0.0855 (0.0187)
XGBoost^e^	0.3155 (0.0061)	0.3223 (0.0190)	0.2476 (0.0182)
RF^f^	0.2962 (0.0065)	0.3116 (0.0146)	0.2717 (0.0302)
Ridge regression	0.3693 (0.0049)	0.3516 (0.0195)	0.1789 (0.0221)

**Table 4:** Response length prediction performance of the compared models.

Model	MAE^a^, mean (SD)	MSE^b^, mean (SD)	*R*^2^, mean (SD)
LightGBM^c^	0.6988 (0.0079)	0.8367 (0.0155)	0.2766 (0.0221)
SVM^d^	0.6941 (0.0054)	0.8905 (0.0149)	0.2302 (0.0145)
XGBoost^e^	0.7219 (0.0072)	0.8688 (0.0168)	0.2490 (0.0129)
RF^f^	0.7058 (0.0069)	0.8637 (0.0146)	0.2532 (0.0218)
Ridge regression	0.7171 (0.0070)	0.8804 (0.0161)	0.2390 (0.0154)

^a^MAE: mean absolute error.

^b^MSE: mean squared error.

^c^LightGBM: Light Gradient Boosting Machine.

^d^SVM: Support Vector Machine.

^e^XGBoost: Extreme Gradient Boosting.

^f^RF: random forest.

### Model Interpretability

#### Global Interpretability

**Figure 6:**
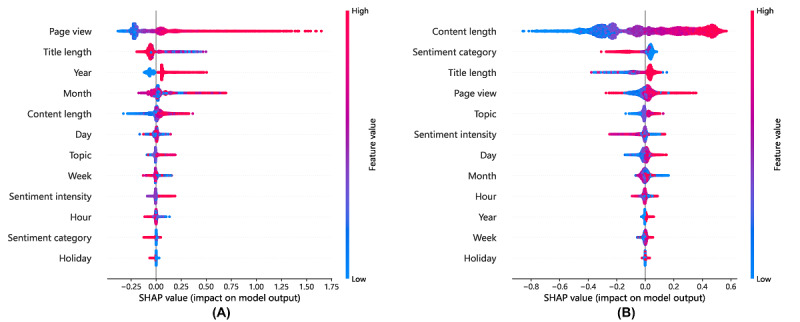
Summary plots of Light Gradient Boosting Machine. (A) Prediction for response quantity. (B) Prediction for response length. SHAP: Shapley Additive Explanations.

We applied the SHAP method to the LightGBM model. The global interpretation graphs of LightGBM for predicting the number of replies and the length of replies are shown in Figure 6. The average SHAP value for each feature is detailed in Multimedia Appendix 5. When a SHAP value of 0 is used as the dividing line, the points on the left indicate the features contributing negatively to the prediction, whereas the points on the right indicate positive contributions. The relationship between each feature and the prediction of the number of replies is shown in Figure 6A. This indicates that positively correlated features include page views, content length, and sentiment intensity. Higher values of these features correspond to a greater number of responses received by the posts. The relationship between each feature and the prediction of response length is shown in Figure 6B. These findings indicate that the length of the question content has a positive effect on response length. Most other features are categorical variables, whose effects are not clearly discernible from the figure.

#### Local Interpretability in Response Quantity Prediction

**Figure 7:**
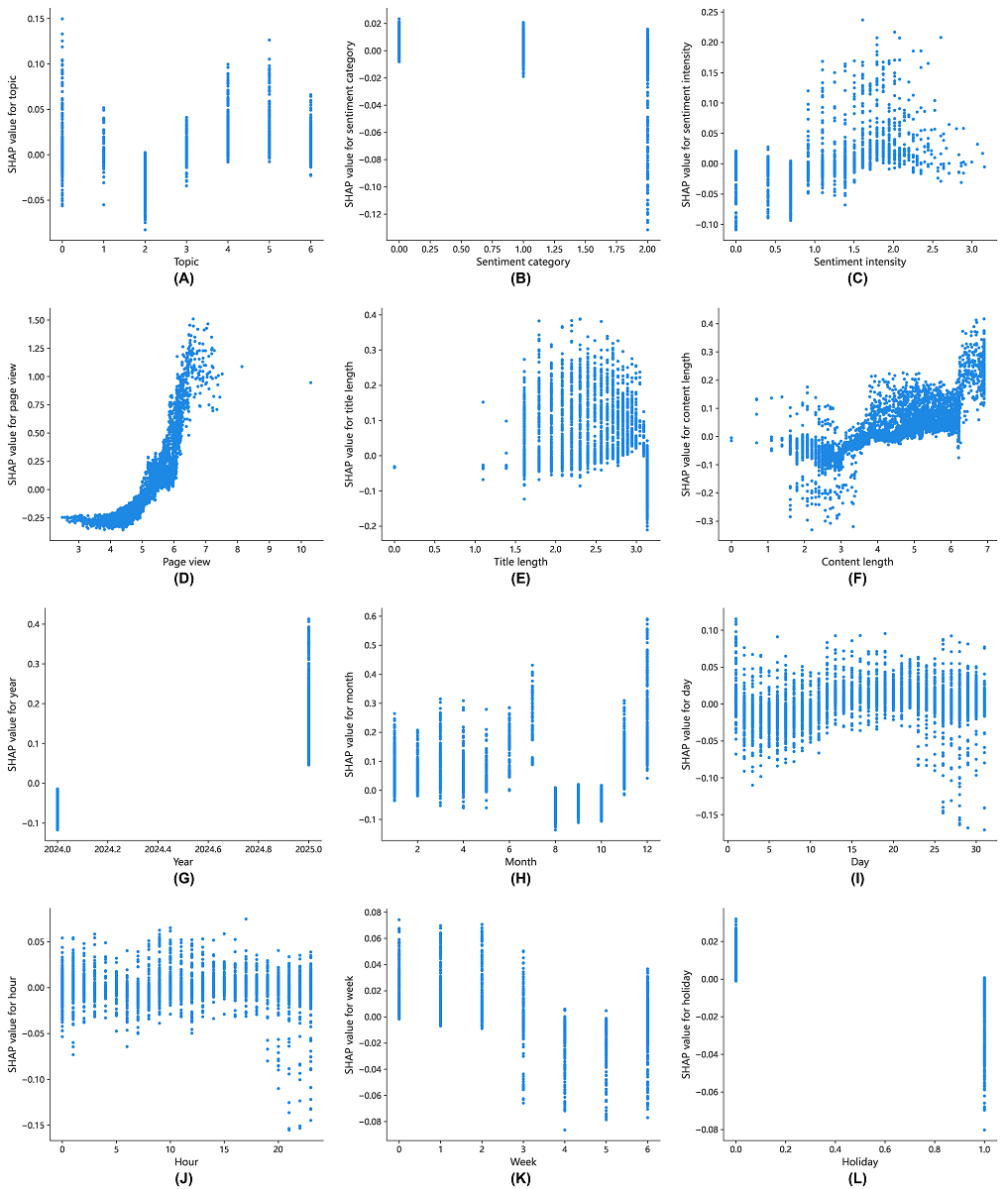
Shapley Additive Explanations (SHAP) dependence plots of Light Gradient Boosting Machine for predicting response quantity. Panels A-L respectively show the SHAP value distributions for the following features: topic, sentiment category, sentiment intensity, page views, title length, content length, year, month, day, hour, week, and holiday.

We constructed feature-SHAP value scatter plots for each feature to analyze its impact on the response quantity (Figure 7). Each dot in the scatter plot represents a single post in our dataset. They illustrate the relationship between feature values (x-axis) and their corresponding SHAP values (y-axis). The SHAP value is a direct measure of how much that specific feature value pushed the model’s prediction toward receiving more (positive SHAP value) or fewer (negative SHAP value) replies. These plots answer a critical question: how a specific post characteristic influences a consultant’s likelihood to reply, and whether this influence is consistently positive, negative, or more complex?

The impact of topic features on response quantity is illustrated in Figure 7A. Topic 2 (depression) has a SHAP value less than 0, whereas topics 3-6 (3: boyfriends or girlfriends; 4: school; 5: marriage; and 6: family) have SHAP values greater than 0. These findings suggest that the topic of depression has a negative effect on response quantity, whereas those related to boyfriends or girlfriends, school, marriage, and family have positive effects. The impact of other topics on response quantity is not clearly defined.

The relationships between post sentiment and response quantity are shown in Figures 7B and 7C. The SHAP values for negative sentiment are greater than 0, indicating a positive contribution to response quantity. In contrast, the SHAP values for positive sentiment are less than 0, indicating a negative contribution. Moreover, when the sentiment intensity exceeds the threshold of 1.7 (equivalent to a sentiment intensity of 5.5), the post sentiment contributes positively to the response quantity. These findings suggest that mental health professionals are more likely to respond to posts that express negative emotions and provide support to high-risk patient users.

The effects of other features on the prediction of response quantity are presented in Figures 7D-7L. The results indicate that when page views exceed the threshold of 5.5 (approximately 244 views), the title length is between 2.75 and 2.95 (approximately 15 to 20 characters), and the content length exceeds the threshold of 4.85 (approximately 127 characters), the posts are more likely to receive professional responses. With respect to the posting time, when the post is published between December and February, in the middle of the month (days 18 to 21), during the period between Monday and Thursday, and on nonpublic holidays, it is more likely to receive a professional response. Moreover, when the page view is less than 5.5 (approximately 244 views), the title length exceeds 2.95 (approximately 20 characters), or when the post is published from August to October, on weekends, or on public holidays, the SHAP values are less than 0, indicating an inhibitory effect on the prediction of response quantity. Additionally, the posting time (hour of day) does not have a significant effect.

#### Local Interpretability in Response Length Prediction

**Figure 8:**
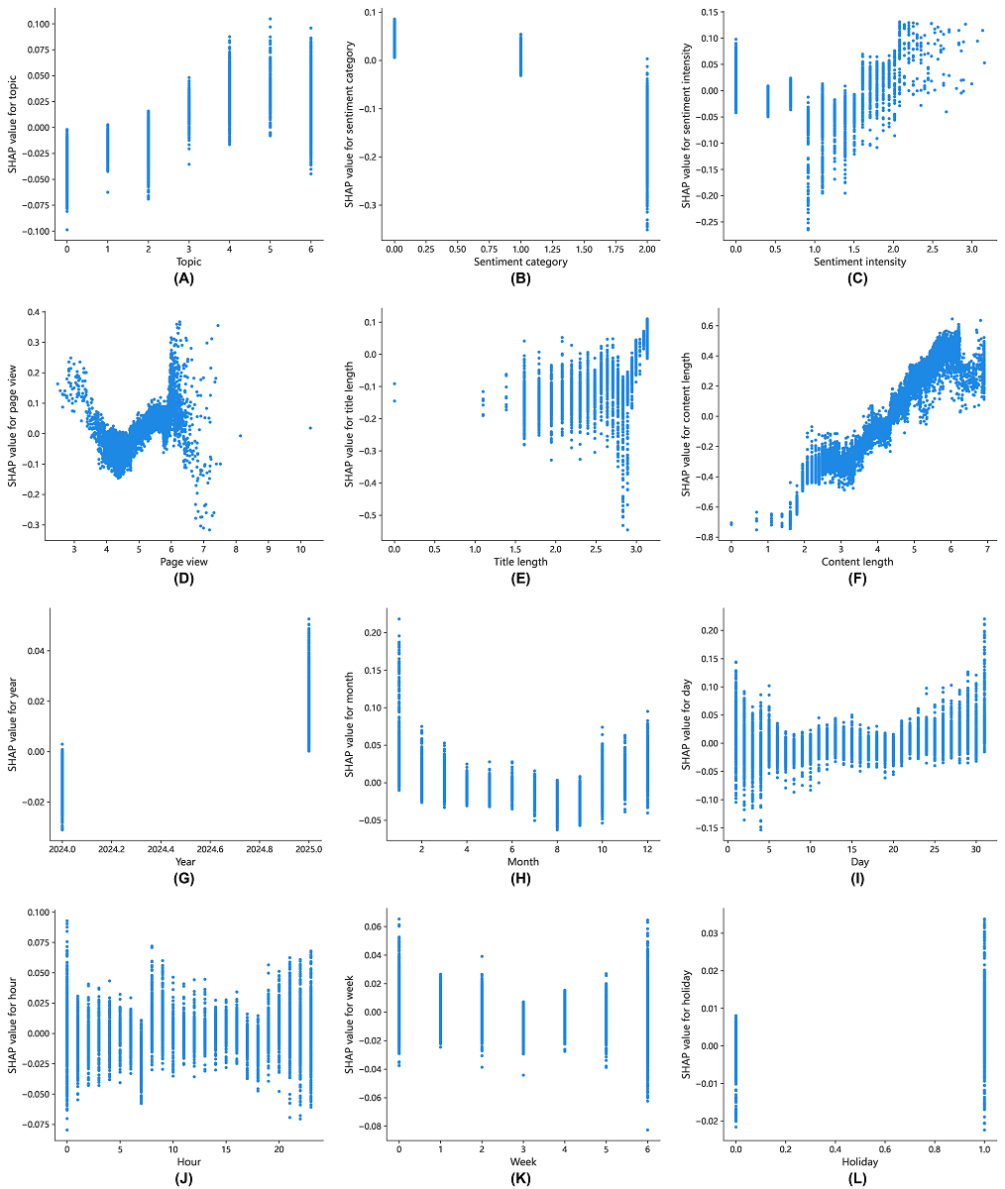
Shapley Additive Explanations (SHAP) dependence plots of Light Gradient Boosting Machine for predicting response length. Panels A-L respectively show the SHAP value distributions for features: topic, sentiment category, sentiment intensity, page views, title length, content length, year, month, day, hour, week, and holiday.

We report SHAP dependence plots for each feature to explain the response length predicted by the LightGBM model (Figure 8). According to Figure 8A, the topic of love has a negative effect on the prediction of response length. The results from Figures 8B and 8C show that negative sentiment has a positive effect on predicting response length, whereas positive emotions have an inhibitory effect. When the sentiment intensity is less than 1.5 (equivalent to the original sentiment intensity of 4.5), it negatively affects the prediction of the reply length. As shown in Figure 8E, when the length of the post’s title is less than the threshold of 2.0 (approximately 7 characters), the predicted response length decreases. As shown in Figure 8F, the critical value for content length is 4.1 (approximately 60 characters), and posts with lengths exceeding 60 characters are more likely to receive longer responses.

### Robustness Analysis

In the robustness analysis, we collected and analyzed forum data from another online mental health platform, YiXinli [52], covering the period from December 2024 to July 2025, with the aim of examining the generalizability of the model interpretations through cross-platform comparison. All the analytical procedures were kept consistent with those applied to the primary dataset. Multimedia Appendix 6 reports the descriptive statistics, topic and sentiment evolution results, model evaluation results, and SHAP-based interpretation outcomes. The findings indicate that the topic distributions, sentiment distributions, and their temporal trends are consistent with those observed on the YiDianLing platform. Moreover, the SHAP interpretations of the LightGBM models for predicting the quantity and length of responses indicate that the effects of key features are relatively consistent with those observed in the primary dataset. These results provide robust support for our findings.

## Discussion

### Principal Findings

This study uses interpretable machine learning techniques to analyze question-and-answer posts from a nonprofit OMHC. The findings highlight the pivotal role of various features of the posts in shaping professionals’ contribution behaviors. First, patient users’ demands for psychological services primarily fall into 7 topic categories: work, love, depression, boyfriends or girlfriends, school, marriage, and family. The majority of user posts are related to depression, which aligns with previous research [14,53]. Users also frequently express concerns related to daily life, work stress, and social relationships [54]. Posts with greater response volumes are often associated with themes such as boyfriends or girlfriends, school, marriage, and family, whereas depression-related posts receive fewer replies. One possible explanation is that depression-related posts are more likely to be posted by diagnosed patients. The forum’s professionals are composed mainly of psychological consultants who are not therapists capable of providing clinical treatment. Therefore, they tend to be more cautious when addressing depression-related issues, thus avoiding the risk of inadvertently harming these patient users. This aligns with prior findings that, in the absence of sufficient knowledge about the individual, consultants may avoid overreacting to questions involving illness and emotions [55].

Second, our study revealed that patient posts with stronger negative emotions are more likely to receive social support in nonprofit mental health communities. From the perspective of social support theory, emotional disclosure by patient users in online communities is crucial for fostering social interaction and obtaining support. Expressions of negative emotions may signal users’ distress, which may elicit empathy and supportive reactions from psychological counselors [56]. On the other hand, emotional intensity can be viewed as an indicator of patients’ level of self-disclosure [57,58]. Intense self-disclosure may enhance users’ motivation to engage [59]. Our analysis shows, for example, that when sentiment intensity is relatively high (eg, above 5.5), posts are more likely to attract longer replies. In contrast, when sentiment intensity is relatively low (eg, less than 4.5), posts tend to receive shorter responses. Drawing on these findings, community managers could provide patient users with emotion-related keywords or tags, which can be automatically selected when writing posts. These emotion-related keywords or tags may enhance users’ ability to articulate their emotional states.

Third, our findings indicate that the title length and content length of patient users’ posts positively influence the quantity and length of professionals’ responses. Longer titles and content may contain greater amounts of information, facilitating better understanding by other community members. Therefore, the amount of information provided in posts positively influences the quality of replies [24]. Our analysis revealed that titles with lengths between 15 and 20 characters and contents with lengths of at least 60 characters attracted more and longer responses. In contrast, titles shorter than approximately 7 characters tend to negatively impact the response length. On the basis of these findings, platform designers may provide real-time feedback on the informativeness of titles and content to post writers. Additionally, the platform can offer high-quality example posts for users to enhance the expressiveness of their posts, thus increasing their likelihood of receiving a response.

In our study, post features, including view count, posting time, day of the week, and public holiday status, influence professional responses heterogeneously. Posts with more than 244 views are associated with greater response volumes. This suggests that posts with greater exposure may attract increased professional participation. Posts generated from Monday to Thursday and on nonpublic holidays receive more responses. Because most of the certified counselors on the platform are not full-time clinical physicians working in offline institutions, they tend to be more active and willing to respond online during weekdays. Our results also show that page views and posting time do not affect response length. One possible explanation is that response length may depend on the professionalism, empathy, and motivation of health professionals rather than on the view number or posting time.

### Research Implications

#### Theoretical Implications

This study deepens our understanding of knowledge contribution behaviors in nonprofit OMHCs. By examining how textual and contextual features of user posts influence the quantity and length of responses from mental health professionals, the findings reveal the important factors that shape health professionals’ knowledge contributions. In addition, this research introduces interpretable machine learning methods into online mental health. This approach addresses the limitations of traditional regression models and black-box algorithms in explaining the influencing mechanisms. It also provides technical support for a deeper understanding of the factors affecting professional response quantity and length.

#### Practical Implications

The findings provide practical guidance for community managers. First, managers can categorize post topics to facilitate precise responses from professionals and help other users explore topics of interest. Second, providing predefined emotion-related keywords or tags on the post editing page helps enhance patients’ ability to express their emotions and may increase the likelihood of receiving a response. Third, providing feedback on information richness and high-quality post templates may help users improve their expression. Fourth, considering the effects of view counts and posting times, platform operators can optimize content visibility strategies. For example, posts with lower view counts and those published during off-peak hours (eg, late night) can be prioritized. This may balance the exposure across posts of varying popularity and publication times and ensure that these posts receive professional responses. This may also enhance the overall fairness and quality of community interactions. Fifth, platform operators must exercise caution when implementing certain strategies (eg, real-time feedback on content informativeness), as encouraging longer posts or greater emotional intensity may inadvertently increase the potential psychological burden on patients. To this end, when designing web interfaces, platforms should position these tools as supplementary and optional. It is crucial to ensure users retain control over these functionalities to balance interaction efficiency with psychological safety.

This study, based on Chinese OMHCs, may offer insights for mental health service platforms in other countries. However, we explicitly caution against the direct generalization of our findings to other cultural contexts. The counselor-led, nonprofit forums of the YiDianLing and YiXinLi platforms embed specific sociotechnical norms, such as text-based communication, work culture, and community governance rules. These factors may shape how patient users present their problems and how consultants perceive their role. Therefore, our conclusions should be interpreted as context-specific insights that highlight the need for future research to “unpack” these contextual differences through comparative studies.

In developing our model, we carefully addressed potential sources of bias, such as by using cross-temporal and cross-platform sampling methods to balance both positive and negative scenarios in predictions. When this research is extended to other countries, adjustments need to be made according to local cultural norms and service systems.

### Limitations and Future Directions

Although our study offers significant contributions, it also has several limitations. First, the data were obtained primarily from the Chinese mental health platform YiDianLing, with supplementary data from YiXinLi used for robustness checks. Given the potential cultural, platform, and user differences across countries, generalizing our findings would require validation using multinational data from diverse platforms. Second, this study uses publicly available posts and response data from OMHCs. Future research could incorporate multimodal data (eg, images and emojis) to gain a deeper understanding of the interactions between patient users and mental health professionals in nonprofit mental health forums. Third, owing to privacy policies, user-level demographics (eg, sex, age, and membership duration) were unavailable. Subsequent studies or online experiments should examine how such characteristics influence forum participation. Fourth, this study solely explores response quantity and length. However, a complete understanding of community interaction also depends on the quality of responses, a multifaceted construct that encompasses aspects like relevance, empathy, and supportiveness. Future work should prioritize developing validated metrics for response quality to better evaluate professional contribution patterns. Furthermore, this study relies on a LightGBM model interpreted with the SHAP method to analyze post feature importance. Given the limited sample size and post-level features, the predictive accuracy may be limited. Future research should thus incorporate richer predictive features and additional interpretable machine learning techniques (eg, Local Interpretable Model-agnostic Explanations) to validate and extend these insights.

### Conclusion

This study uses explainable machine learning methods to investigate the post features that influence response quantity and length in OMHCs. It highlights the importance of the post topic, post title, post length, post sentiment, and posting time. These findings provide insights for platform managers in terms of optimizing functional design and improving the effectiveness of community interactions.

## Appendix

Multimedia Appendix 1 Temporal dynamics of posts and responses on the YiDianLing platform.

Multimedia Appendix 2 Model performance evaluation.

Multimedia Appendix 3 The hyperparameter settings for the Shapley Additive Explanations algorithm.

Multimedia Appendix 4 Representative keywords for each topic identified by BERTopic.

Multimedia Appendix 5 Average absolute Shapley Additive Explanations values of features for predicting response quantity and length.

Multimedia Appendix 6 Analysis results for the YiXinli platform.

## Abbreviations

BERT: Bidirectional Encoder Representations from Transformers
DistilBERT: distilled Bidirectional Encoder Representations from Transformers
LightGBM: Light Gradient Boosting Machine
OMHC: online mental health community
RF: random forest
SHAP: Shapley Additive Explanations
SVM: Support Vector Machine
TF-IDF: term frequency-inverse document frequency
XGBoost: Extreme Gradient Boosting

## Data Availability

The data used in this study are available upon reasonable request from the corresponding author.

## References

[1] De Hert M, Detraux J, Vancampfort D (2018). The intriguing relationship between coronary heart disease and mental disorders. Dialogues Clin Neurosci.

[2] Beurel E, Toups M, Nemeroff CB (2020). The bidirectional relationship of depression and inflammation: double trouble. Neuron.

[3] Ohrnberger J, Fichera E, Sutton M (2017). The relationship between physical and mental health: a mediation analysis. Soc Sci Med.

[4] Drissi N, Ouhbi S, Janati Idrissi MA, Fernandez-Luque L, Ghogho M (2020). Connected mental health: systematic mapping study. J Med Internet Res.

[5] Schuch FB, Vancampfort D (2021). Physical activity, exercise, and mental disorders: it is time to move on. Trends Psychiatry Psychother.

[6] Walker ER, McGee RE, Druss BG (2015). Mortality in mental disorders and global disease burden implications: a systematic review and meta-analysis. JAMA Psychiatry.

[7] Ali K, Farrer L, Gulliver A, Griffiths KM (2015). Online peer-to-peer support for young people with mental health problems: a systematic review. JMIR Ment Health.

[8] Li Pira G, Aquilini B, Davoli A, Grandi S, Ruini C (2023). The use of virtual reality interventions to promote positive mental health: systematic literature review. JMIR Ment Health.

[9] Tzeng Y, Yin W, Lin K, Wei J, Liou H, Sung H, Lang H (2022). Factors associated with the utilization of outpatient virtual clinics: retrospective observational study using multilevel analysis. J Med Internet Res.

[10] Oexle N, Ajdacic-Gross V, Kilian R, Müller M, Rodgers S, Xu Z, Rössler W, Rüsch N (2017). Mental illness stigma, secrecy and suicidal ideation. Epidemiol Psychiatr Sci.

[11] Banwell E, Hanley T, De Ossorno Garcia S, Mindel C, Kayll T, Sefi A (2022). The helpfulness of web-based mental health and well-being forums for providing peer support for young people: cross-sectional exploration. JMIR Form Res.

[12] Liu J, Gao L (2022). Lurking or active? The influence of user participation behavior in online mental health communities on the choice and evaluation of doctors. J Affect Disord.

[13] Park A, Conway M, Chen AT (2018). Examining thematic similarity, difference, and membership in three online mental health communities from reddit: a text mining and visualization approach. Comput Human Behav.

[14] Feldhege J, Moessner M, Bauer S (2020). Who says what? Content and participation characteristics in an online depression community. J Affect Disord.

[15] Saha B, Nguyen T, Phung D, Venkatesh S (2016). A framework for classifying online mental health-related communities with an interest in depression. IEEE J Biomed Health Inform.

[16] Grub MF (2025). Reddit as a "Safe Space": topic modeling of online mental health communities for depression and anxiety. Weizenbaum J Digit Soc.

[17] AbouWarda H, Miscione G (2025). Understanding how discourse themes in an online mental health community on twitter/x drive varied population-specific empowerment processes in alignment with global standards: a qualitative analysis of #bipolarclub. J Med Internet Res.

[18] Zhou J, Zuo M, Ye C (2019). Understanding the factors influencing health professionals' online voluntary behaviors: evidence from YiXinLi, a Chinese online health community for mental health. Int J Med Inform.

[19] Kim M, Saha K, De Choudhury M, Choi D (2023). Supporters First: understanding online social support on mental health from a supporter perspective. Proc ACM Hum-Comput Interact.

[20] Liu S, Xiao W, Fang C, Zhang X, Lin J (2020). Social support, belongingness, and value co-creation behaviors in online health communities. Telemat Inform.

[21] Magane KM, Kenney M, Nelson E, Wisk L, Weitzman ER (2017). The quality and safety of online health communities engaging adolescents around depression and substance use: a multisite evaluation. J Adolesc Health.

[22] Marshall P, Booth M, Coole M, Fothergill L, Glossop Z, Haines J, Harding A, Johnston R, Jones S, Lodge C, Machin K, Meacock R, Nielson K, Puddephatt J, Rakic T, Rayson P, Robinson H, Rycroft-Malone J, Shryane N, Swithenbank Z, Wise S, Lobban F (2024). Understanding the impacts of online mental health peer support forums: realist synthesis. JMIR Ment Health.

[23] Morini V, Sansoni M, Rossetti G, Pedreschi D, Castillo C (2025). Participant behavior and community response in online mental health communities: insights from reddit. Computers in Human Behavior.

[24] Li J, Liu D, Wan C, Liang Z, Zhu T (2023). Empirical study of factors that influence the perceived usefulness of online mental health community members. Psych J.

[25] Smith-Merry J, Goggin G, Campbell A, McKenzie K, Ridout B, Baylosis C (2019). Social connection and online engagement: insights from interviews with users of a mental health online forum. JMIR Ment Health.

[26] Sharma E, De Choudhury CM (2018). Mental health support and its relationship to linguistic accommodation in online communities.

[27] Srivastava A, Gupta T, Cerezo A, Lord SP, Akhtar MS, Chakraborty T (2025). Critical behavioral traits foster peer engagement in online mental health communities. PLoS One.

[28] Chen L, Baird A, Straub D (2020). A linguistic signaling model of social support exchange in online health communities. Decis Support Syst.

[29] Wang J, Chiu Y, Yu H, Hsu Y (2017). Understanding a nonlinear causal relationship between rewards and physicians' contributions in online health care communities: longitudinal study. J Med Internet Res.

[30] Zhou T (2021). Examining online health community users’ sharing behaviour: a social influence perspective. Inf Dev.

[31] Chen Q, Jin J, Yan X (2022). Understanding physicians' motivations for community participation and content contribution in online health communities. OIR.

[32] Imlawi J, Gregg D (2020). Understanding the satisfaction and continuance intention of knowledge contribution by health professionals in online health communities. Inform Health Soc Care.

[33] Maheshwari B, Sarrion M, Motiani M, O'Sullivan S, Chandwani R (2020). Exploration of factors affecting the use of Web 2.0 for knowledge sharing among healthcare professionals: an Indian perspective. JKM.

[34] Derks D, Fischer AH, Bos AER (2008). The role of emotion in computer-mediated communication: a review. Comput Hum Behav.

[35] Zhuo X, Wang W (2024). Why are physicians willing to contribute knowledge? Evidence from online health communities. Comput Hum Behav.

[36] Feng X, Hu Y, Pfaff H, Liu S, Xie J, Zhang Z (2024). Exploring client preferences for psychological counselors in a chinese online health community: longitudinal study. J Med Internet Res.

[37] Liu H, Zhang L, Wang W, Huang Y, Li S, Ren Z, Zhou Z (2022). Prediction of online psychological help-seeking behavior during the COVID-19 pandemic: an interpretable machine learning method. Front Public Health.

[38] Baba A, Bunji K (2023). Prediction of mental health problem using annual student health survey: machine learning approach. JMIR Ment Health.

[39] Huang Y, Liu H, Chi M, Meng S, Wang W (2025). Digit Health.

[40] (2025). YiDianLing.

[41] Devlin J, Chang M, Lee K, Toutanova K (2019). BERT: Pre-training of deep bidirectional transformers for language understanding.

[42] Grootendorst M BERTopic: neural topic modeling with a class-based TF-IDF procedure. arXiv.

[43] (2025). DistilBERT-based multilingual sentiment classification model. Hugging Face.

[44] Sanh V, Debut L, Chaumond J, Wolf T DistilBERT, a distilled version of BERT: smaller, faster, cheaper and lighter. arXiv.

[45] AlQadi RA, Taie SA, Idrees AM, Elhariri E (2025). Explainable deep learning model for ChatGPT-rephrased fake review detection using DistilBERT. BDCC.

[46] Jojoa M, Eftekhar P, Nowrouzi-Kia B, Garcia-Zapirain B (2022). Natural language processing analysis applied to COVID-19 open-text opinions using a distilBERT model for sentiment categorization. AI Soc.

[47] Ke G, Meng Q, Finley T, Wang T, Chen W, Ma W, Ye Q (2017). LightGBM: a highly efficient gradient boosting decision tree.

[48] Tai C, Liao T, Chen S, Chung M (2024). Sleep stage classification using light gradient boost machine: exploring feature impact in depressive and healthy participants. Biomed Sigal Process Control.

[49] (2025). LightGBM parameters tuning. Read the Docs.

[50] Le Glaz A, Haralambous Y, Kim-Dufor D, Lenca P, Billot R, Ryan TC, Marsh J, DeVylder J, Walter M, Berrouiguet S, Lemey C (2021). Machine learning and natural language processing in mental health: systematic review. J Med Internet Res.

[51] Lundberg SM, Erion G, Chen H, DeGrave A, Prutkin JM, Nair B, Katz R, Himmelfarb J, Bansal N, Lee S (2020). From local explanations to global understanding with explainable AI for trees. Nat Mach Intell.

[52] YiXinLi.

[53] González Moreno A, Molero Jurado MDM (2024). Presence of emotions in network discourse on mental health: thematic analysis. Psychiatry Int.

[54] Akar E (2025). Connecting for well-being: a role-based network analysis of online mental health communities. Behav Inf Technol.

[55] Zhang L, Liu D, Li J, Wan C, Liu X (2024). Exploring linguistic features and user engagement in Chinese online mental health counseling. Heliyon.

[56] De Choudhury M, De S (2014). Mental health discourse on reddit: self-disclosure, social support, and anonymity. ICWSM.

[57] Chu TH, Sun M, Crystal Jiang L (2022). Self-disclosure in social media and psychological well-being: A meta-analysis. J Soc Pers Relatsh.

[58] Liu J, Kong J (2021). Why do users of online mental health communities get likes and reposts: a combination of text mining and empirical analysis. Healthcare (Basel).

[59] Liu J, Liu Y (2022). Exploring the user interaction network in an anxiety disorder online community: an exponential random graph model with topical and emotional effects. Int J Environ Res Public Health.

