# Supplementary material for: Predictors of Professional Responses in Nonprofit Mental Health Forums: Interpretable Machine Learning Analysis
Source: J Med Internet Res. 2026 Jan 5;28:e74359. doi: 10.2196/74359 (PMC12817036; doi:10.2196/74359)
Supplement: Multimedia Appendix 1 [file jmir_v28i1e74359_app1.docx]

**Appendix 1. The temporal dynamics chart of the number of posts and received replies on the platform from August 2024 to July 2025.**
