# Supplementary material for: Predictors of Professional Responses in Nonprofit Mental Health Forums: Interpretable Machine Learning Analysis
Source: J Med Internet Res. 2026 Jan 5;28:e74359. doi: 10.2196/74359 (PMC12817036; doi:10.2196/74359)

**Appendix 2. Comparisons of model performance.**

**Figure A2.1** Performance of compared models in predicting response quantity(A) and response length(B)


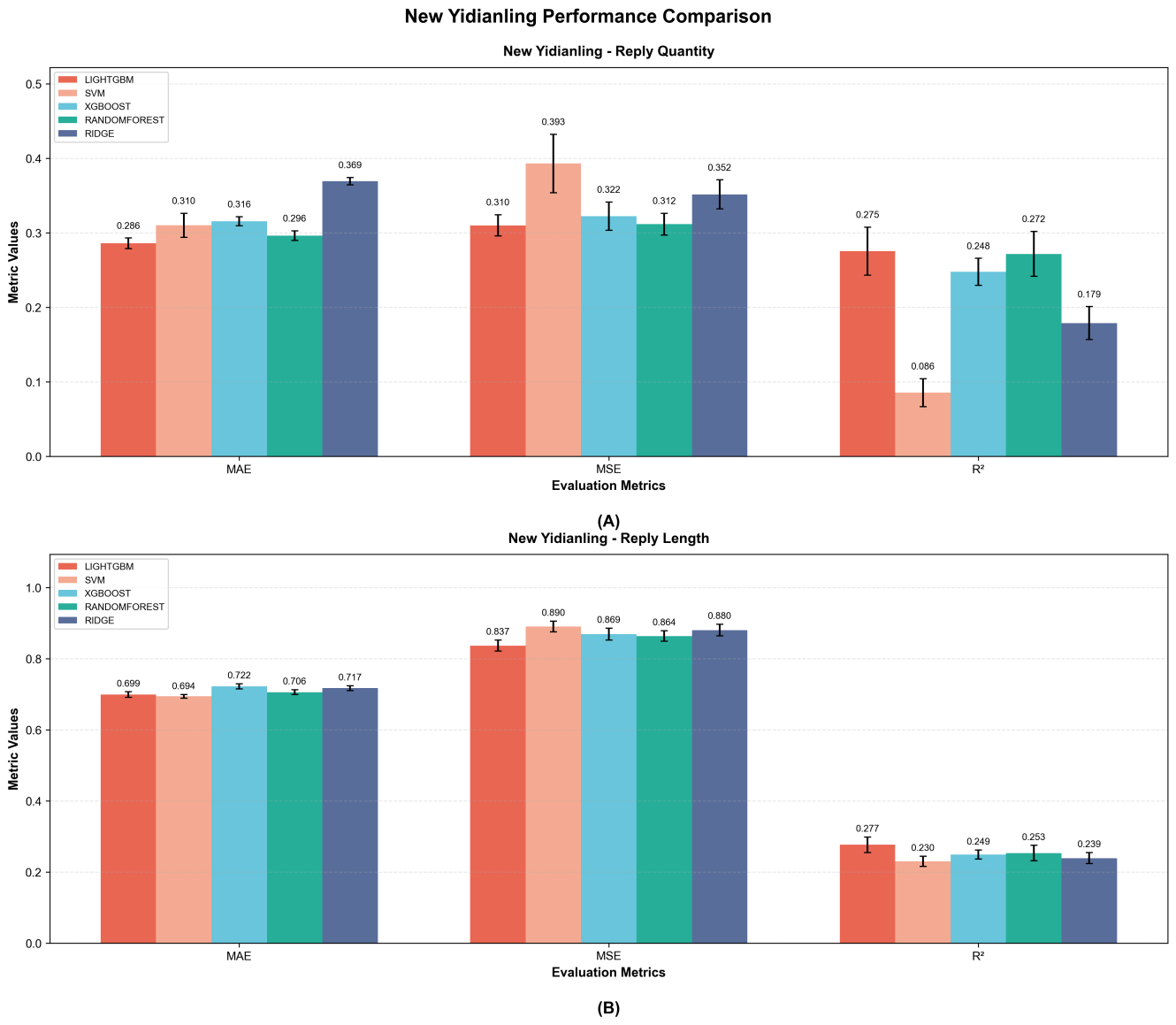


**Figure A2.2** Calibration curves for the LightGBM model in predicting response quantity and length


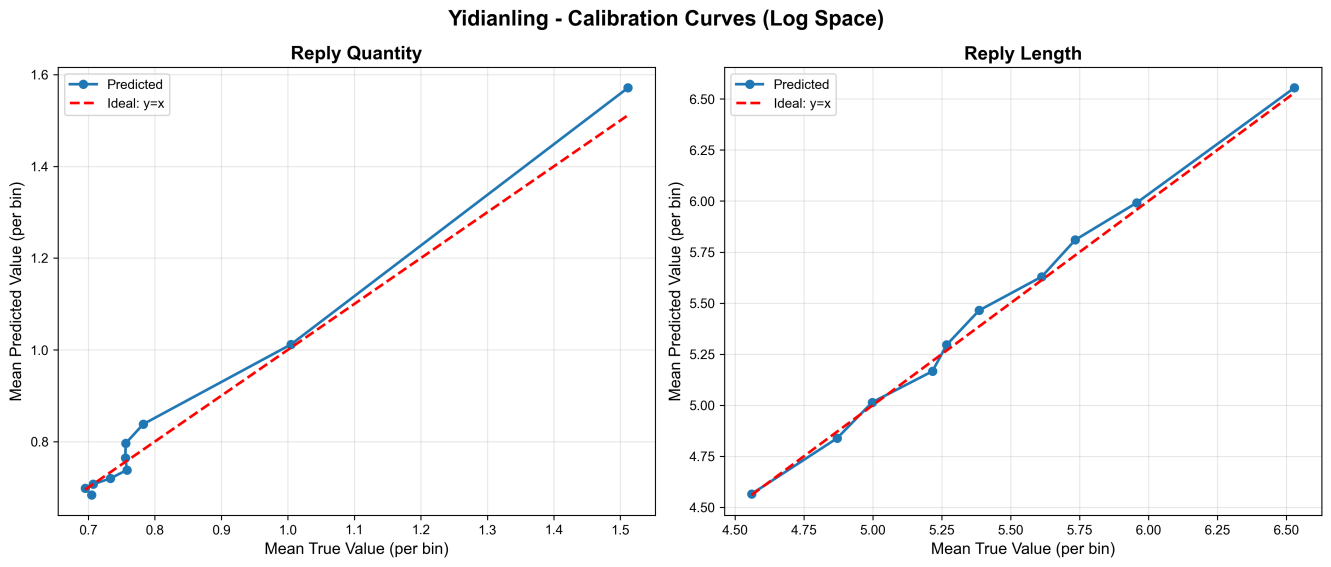

Supplement: Multimedia Appendix 2 [file jmir_v28i1e74359_app2.docx]
