# Supplementary material for: Predictors of Professional Responses in Nonprofit Mental Health Forums: Interpretable Machine Learning Analysis
Source: J Med Internet Res. 2026 Jan 5;28:e74359. doi: 10.2196/74359 (PMC12817036; doi:10.2196/74359)
Supplement: Multimedia Appendix 3 [file jmir_v28i1e74359_app3.docx]

**Appendix 3. The hyperparameters used for LightGBM algorithm.**

| **Hyperparameter** | **Setting value** |
| --- | --- |
| learning_rate | 0.05 |
| num_leaves | 40 |
| max_depth | 6 |
| feature_fraction | 0.8 |
| bagging_fraction | 1 |
| bagging_freq | 5 |
| verbose | 1 |
| min_child_samples | 20 |
| min_child_weight | 0.001 |
| reg_alpha | 0 |
| reg_lambda | 0.03 |
| others | default |
