# Supplementary material for: Predictors of Professional Responses in Nonprofit Mental Health Forums: Interpretable Machine Learning Analysis
Source: J Med Internet Res. 2026 Jan 5;28:e74359. doi: 10.2196/74359 (PMC12817036; doi:10.2196/74359)
Supplement: Multimedia Appendix 4 [file jmir_v28i1e74359_app4.docx]

**Appendix 4. Representative keywords for each topic**

| **Topic 0 Work** | **Topic 1 Love** |
| --- | --- |
| ('工作', 0.07518546313344304),  ('领导', 0.04424045695360732),  ('公司', 0.03759806923351628),  ('同事', 0.034104287138364796),  ('上班', 0.028953502466633453),  ('老板', 0.023162248252684415),  ('辞职', 0.02024312698083411),  ('面试', 0.016136500542144442),  ('工资', 0.015994556766731158),  ('部门', 0.013226208979521089) | ('爱情', 0.08816668269899187),  ('恋爱', 0.04654556588918143),  ('谈恋爱', 0.044231331279530356),  ('感情', 0.024843629941075186),  ('爱上', 0.022849449753882655),  ('爱是', 0.021569332571708743),  ('不爱', 0.021208077572826646),  ('不会', 0.01713853223248127),  ('不是', 0.01712077860978207),  ('没有', 0.015963460760288258) |
| **Topic 2 Depression** | **Topic 3 Boyfriends or girlfriends** |
| ('抑郁症', 0.12644981759446053),  ('抑郁', 0.10776090444847543),  ('重度', 0.041399363215574865),  ('医院', 0.03976760947041658),  ('医生', 0.03316321275668107),  ('中度', 0.030043167792653758),  ('测试', 0.024243638757026738),  ('检查', 0.0223919293537624),  ('吃药', 0.022113275074915634),  ('焦虑', 0.02173554307270668) | ('分手', 0.04913968082526995),  ('男朋友', 0.047291907967112565),  ('男生', 0.02886438547654642),  ('女生', 0.028828612303527935),  ('女朋友', 0.020671044892240583),  ('感情', 0.016366540718654104),  ('聊天', 0.016120569991973414),  ('朋友', 0.015988710239831054),  ('没有', 0.014589050003353607),  ('男友', 0.014025405423164132) |
| **Topic 4 School** | **Topic 5 Marriage** |
| ('老师', 0.03358534322586654),  ('学校', 0.02553727314048331),  ('学习', 0.025215590685333356),  ('同学', 0.02321205785232215),  ('成绩', 0.01915610021976156),  ('学生', 0.015250760704149161),  ('不想', 0.014200228065432583),  ('高中', 0.01382448508080799),  ('考试', 0.013348575706951929),  ('特别', 0.01313713855267273) | ('老公', 0.0669073348539074),  ('结婚', 0.0469177849711366),  ('孩子', 0.039465336318102485),  ('离婚', 0.03891725658299116),  ('婚姻', 0.02171088871506089),  ('老婆', 0.017481733883135744),  ('没有', 0.014863015281785992),  ('婆婆', 0.01433867337271926),  ('出轨', 0.01418950442535707),  ('吵架', 0.014011858882520264) |
| **Topic 6 Family** |  |
| ('妈妈', 0.03463224759687151),  ('父母', 0.026367011950798318),  ('我妈', 0.025720007669481258),  ('孩子', 0.02495444935360891),  ('爸爸', 0.02406740851654039),  ('我爸', 0.019145285440046275),  ('母亲', 0.017365918418750934),  ('父亲', 0.016479102884723974),  ('弟弟', 0.016240294214852114),  ('家里', 0.015008222846458297) |  |
