# Supplementary material for: Predictors of Professional Responses in Nonprofit Mental Health Forums: Interpretable Machine Learning Analysis
Source: J Med Internet Res. 2026 Jan 5;28:e74359. doi: 10.2196/74359 (PMC12817036; doi:10.2196/74359)
Supplement: Multimedia Appendix 5 [file jmir_v28i1e74359_app5.docx]

**Appendix 5. Feature importance based on average absolute SHAP value in LightGBM model.**

**4.1 The average absolute SHAP values of LightGBM features for predicting response quantity.**

| Rank | Variables | Mean \|SHAP\| value |
| --- | --- | --- |
| 1 | Page view | 0.186968 |
| 2 | Title length | 0.072904 |
| 3 | Year | 0.069683 |
| 4 | Month | 0.046171 |
| 5 | Content length | 0.045842 |
| 6 | Day | 0.013406 |
| 7 | Topic | 0.01203 |
| 8 | Week | 0.010663 |
| 9 | Sentiment intensity | 0.010633 |
| 10 | Hour | 0.006638 |
| 11 | Sentiment category | 0.003749 |
| 12 | Holiday | 0.003024 |

**4.2 The average absolute SHAP values of LightGBM features for predicting response length.**

| Rank | Variables | Mean \|SHAP\| value |
| --- | --- | --- |
| 1 | Content length | 0.273548 |
| 2 | Sentiment category | 0.054133 |
| 3 | Title length | 0.053445 |
| 4 | Page view | 0.037769 |
| 5 | Topic | 0.020371 |
| 6 | Sentiment intensity | 0.018535 |
| 7 | Day | 0.017157 |
| 8 | Month | 0.015853 |
| 9 | Hour | 0.010364 |
| 10 | Year | 0.007193 |
| 11 | Week | 0.006646 |
| 12 | Holiday | 0.002651 |
