# Supplementary material for: Predictors of Professional Responses in Nonprofit Mental Health Forums: Interpretable Machine Learning Analysis
Source: J Med Internet Res. 2026 Jan 5;28:e74359. doi: 10.2196/74359 (PMC12817036; doi:10.2196/74359)
Supplement: Multimedia Appendix 6 [file jmir_v28i1e74359_app6.docx]

**Appendix 6. Descriptive statistics, topic and sentiment evolution results, model evaluation results and SHAP interpretation results from the YiXinLi platform.**

**6.1 Descriptive statistics of features**

| **Features** | **N** | **Mean±SD/n(%)** |
| --- | --- | --- |
| **Page view** | 4684 | 5.13±0.86 |
| **Sentiment category** |  |  |
| Positive | 519 | 11.10 |
| Neutral | 723 | 15.40 |
| Negative | 3442 | 73.50 |
| **Sentiment intensity** | 4684 | 0.92±0.65 |
| **Topic** |  |  |
| Work | 479 | 10.20 |
| Love | 30 | 0.60 |
| Depression | 1458 | 31.10 |
| Boyfriends or girlfriends | 26 | 6.30 |
| School | 1000 | 21.30 |
| Marriage | 418 | 8.90 |
| Family | 1003 | 21.40 |
| **Title length** | 4684 | 3.14±0.13 |
| **Content length** | 4684 | 5.21±0.98 |
| **Reply quantity** | 4684 | 1.13±0.93 |
| **Reply quality** | 4684 | 6.54±0.58 |

**6.2 Topic frequency evolution**


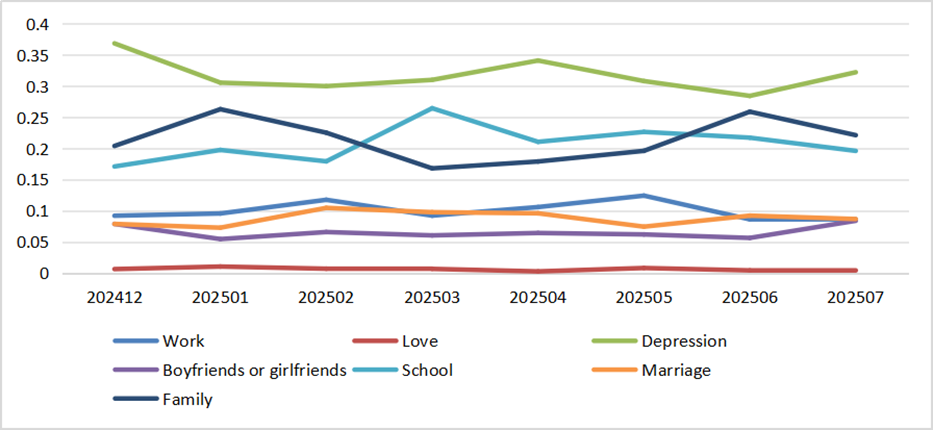


**6.3. Emotional map on different dates**


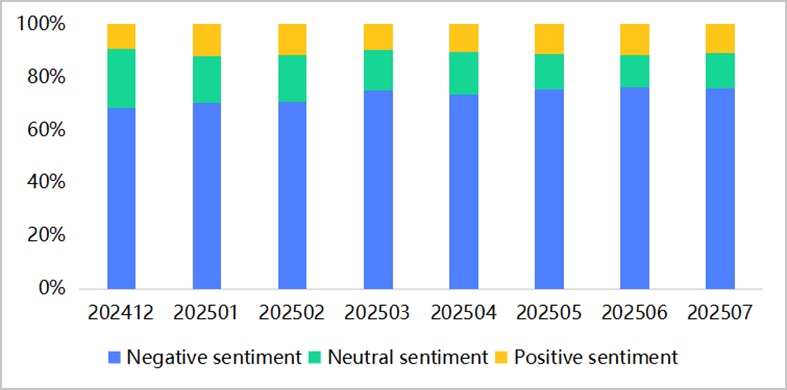


**6.4. Evaluation results of five models in response quantity(A) and response length(B) prediction.**


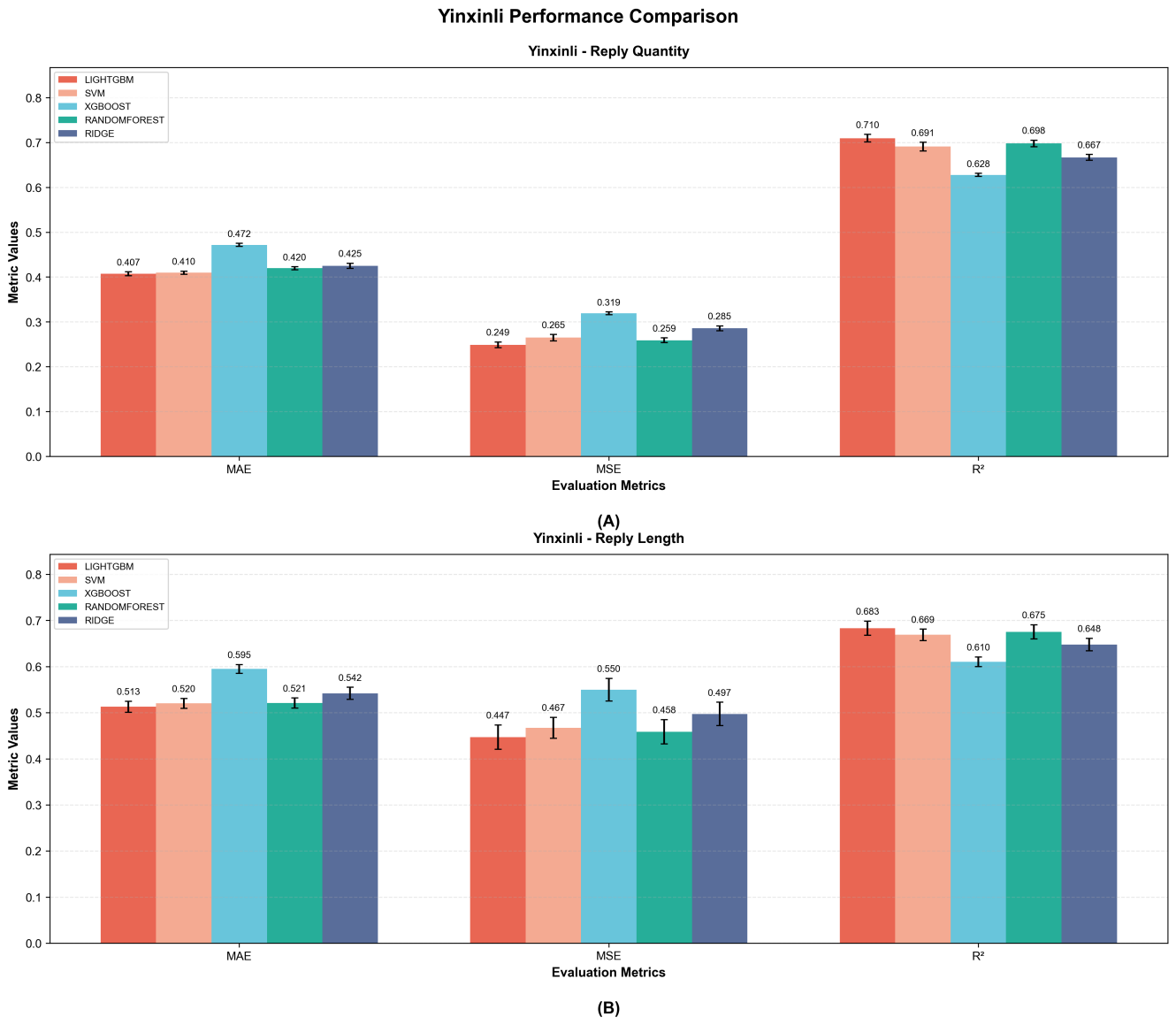


**6.5. Calibration curves for the LightGBM model in response quantity and length prediction tasks.**


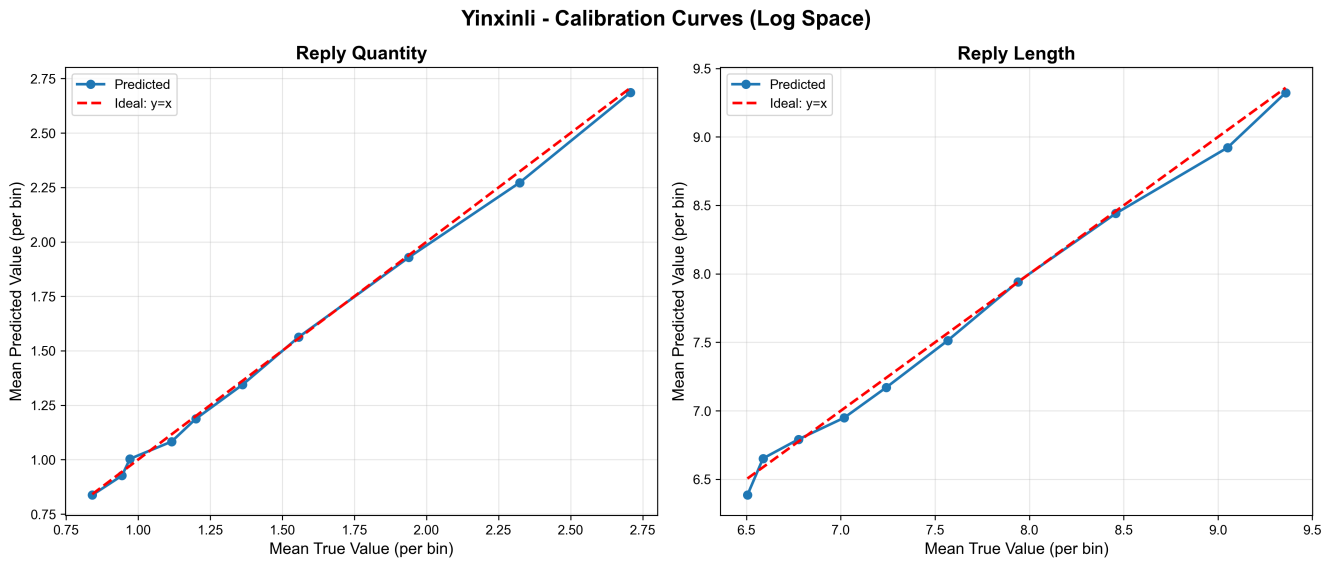


**6.4 Summary plots of LightGBM for predicting response quantity (A) and response quality(B).**

| 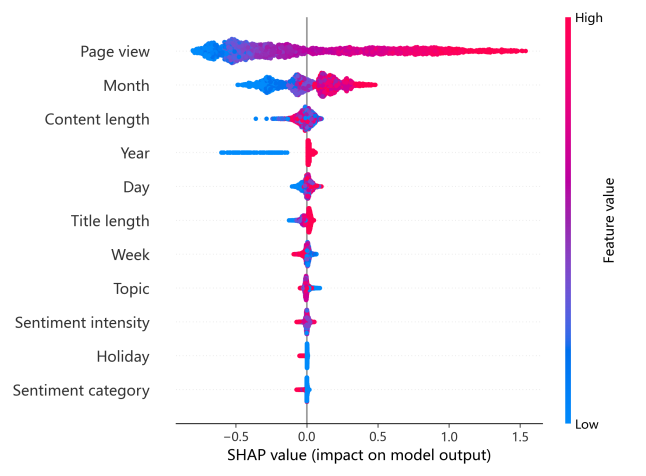 | 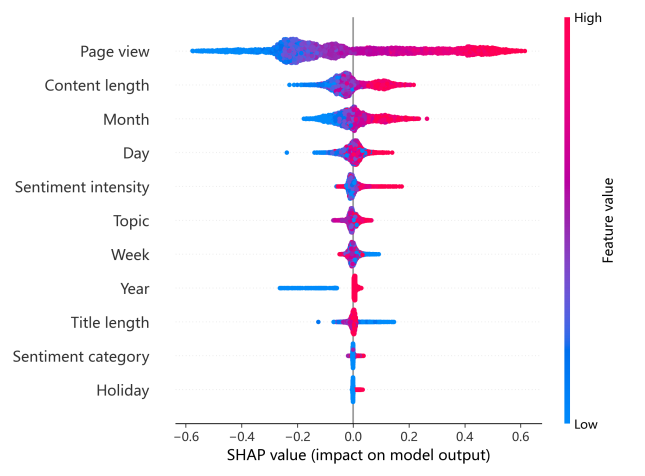 |
| --- | --- |
| (A) | (B) |

**6.5 SHAP dependence plots of LightGBM for predicting response quantity. A-L respectively show the SHAP value distributions for the features: topic, sentiment category, sentiment intensity, page views, title length, content length, year, month, day, week, and holiday.**

| 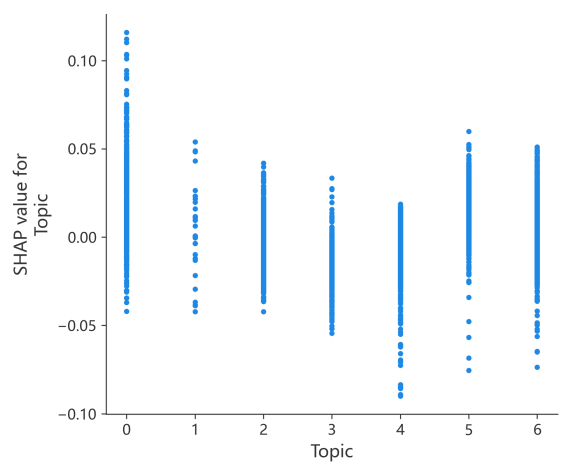 | 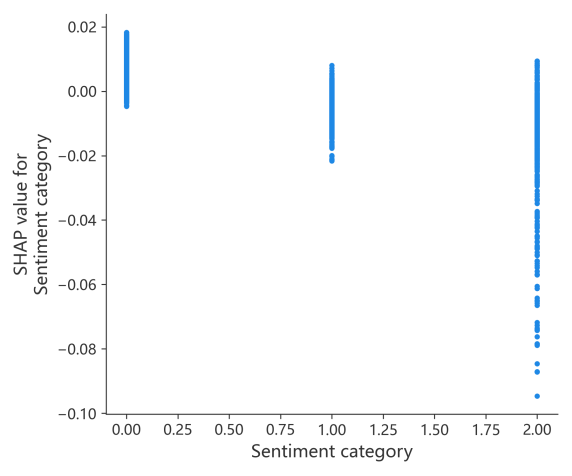 |
| --- | --- |
| A | B |
| 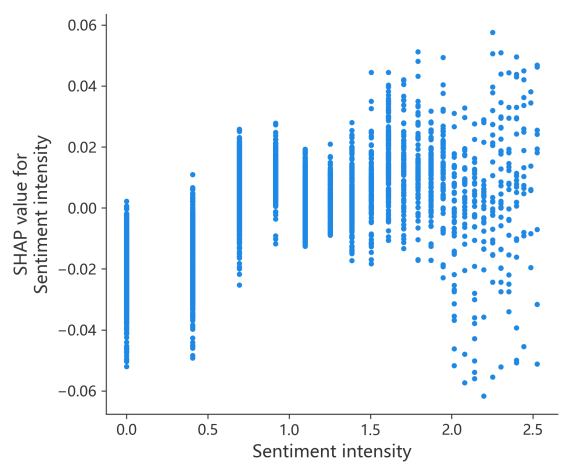 | 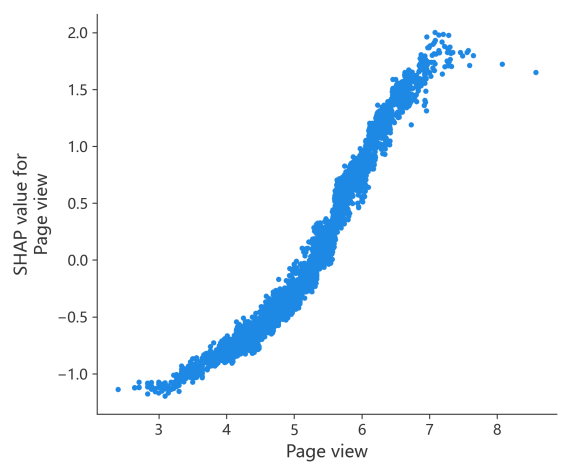 |
| C | D |
| 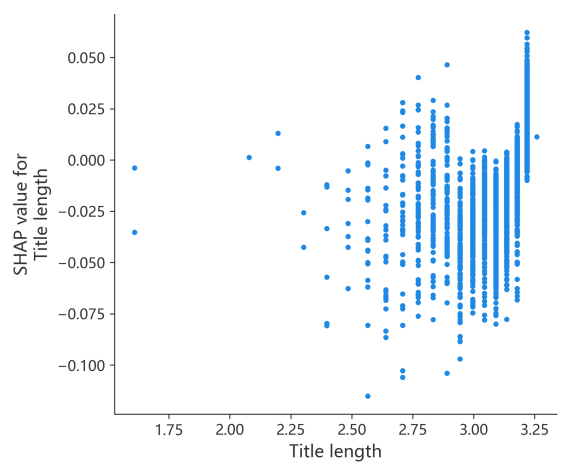 | 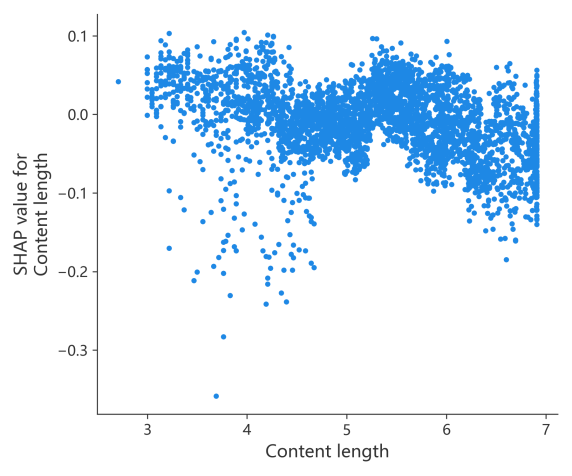 |
| E | F |
| 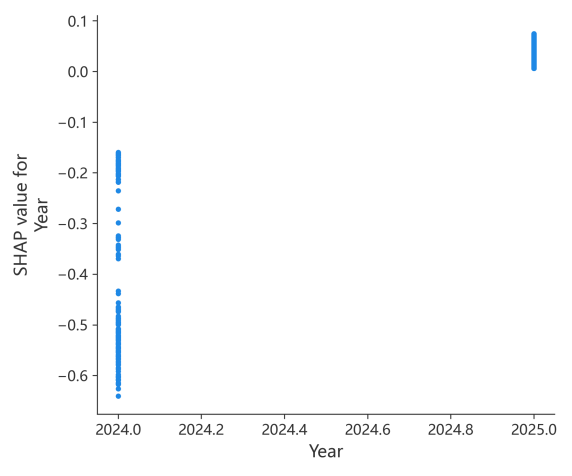 | 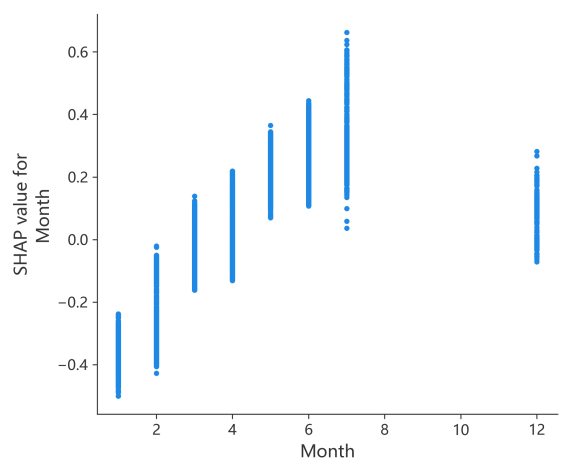 |
| G | H |
| 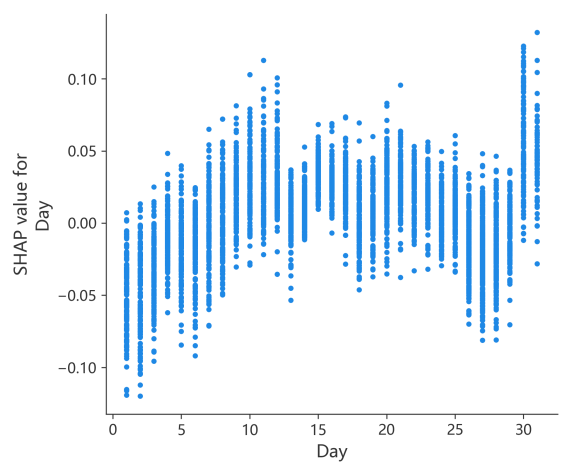 | 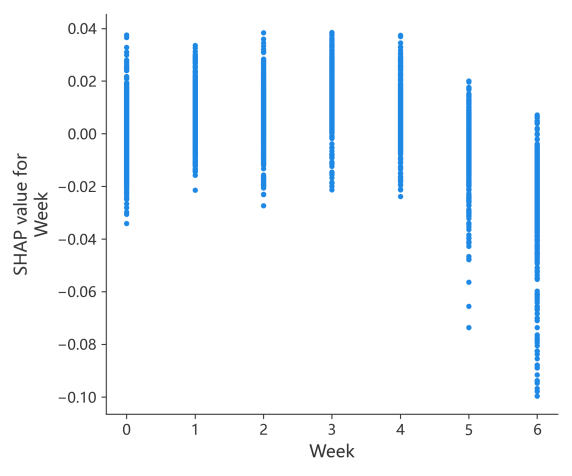 |
| I | J |
| 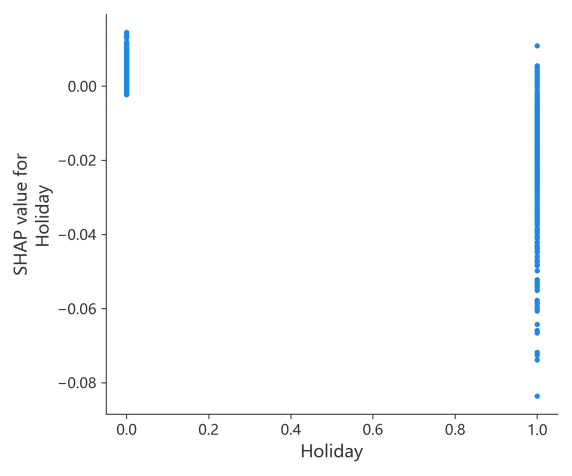 |  |
| K |  |

**6.6 SHAP dependence plots of LightGBM for predicting response quality. A-L respectively show the SHAP value distributions for the features: topic, sentiment category, sentiment intensity, page views, title length, content length, year, month, day, week, and holiday.**

| 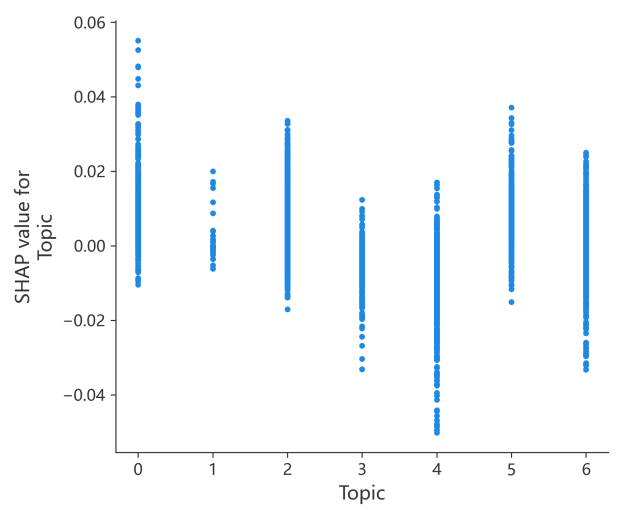 | 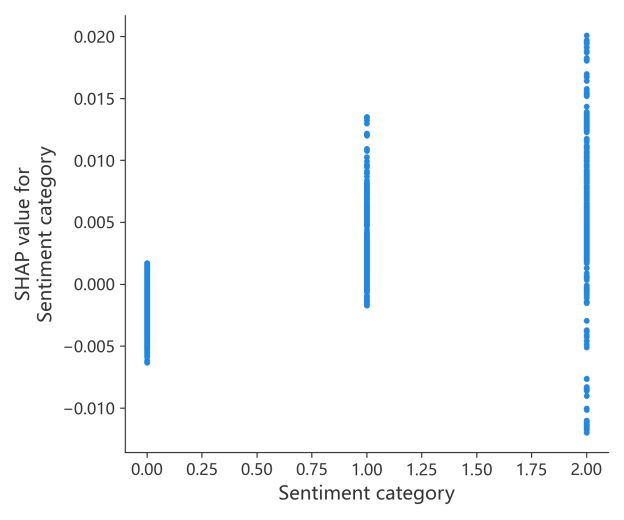 |
| --- | --- |
| A | B |
| 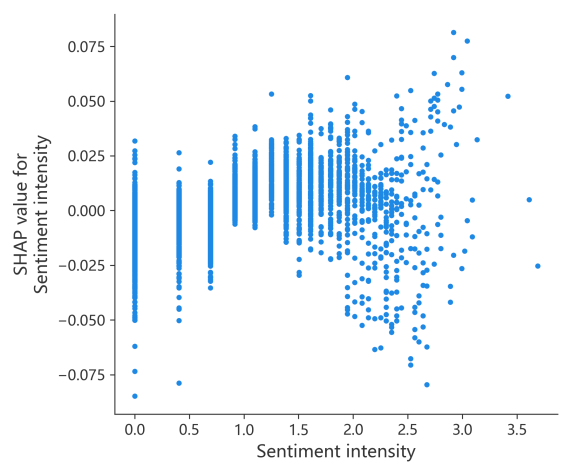 | 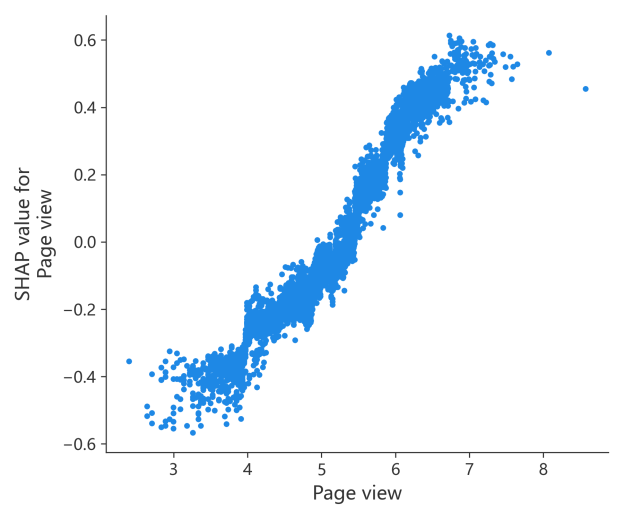 |
| C | D |
| 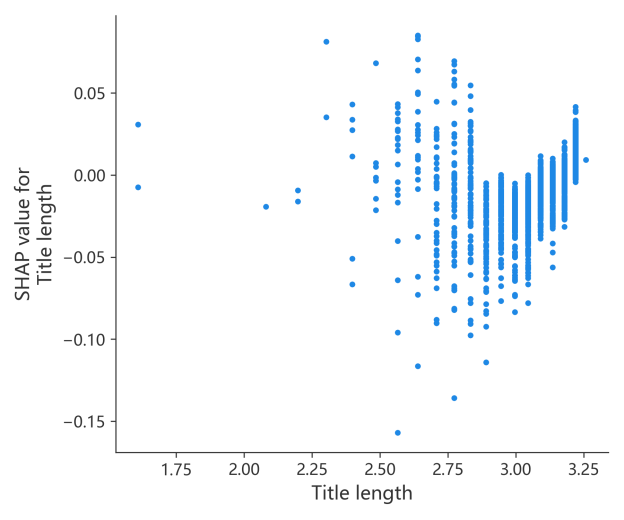 | 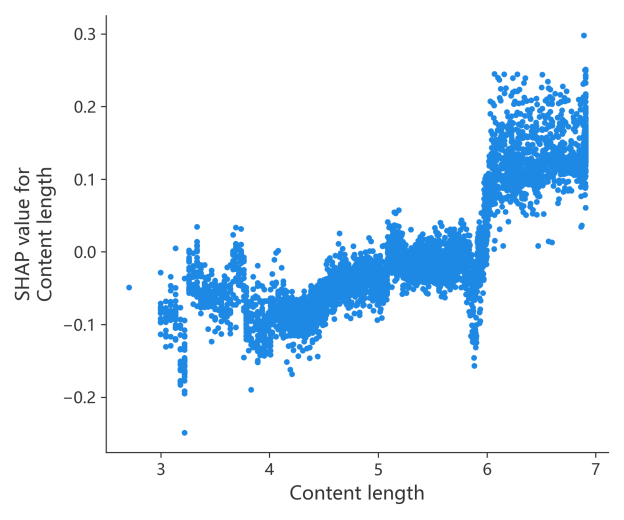 |
| E | F |
| 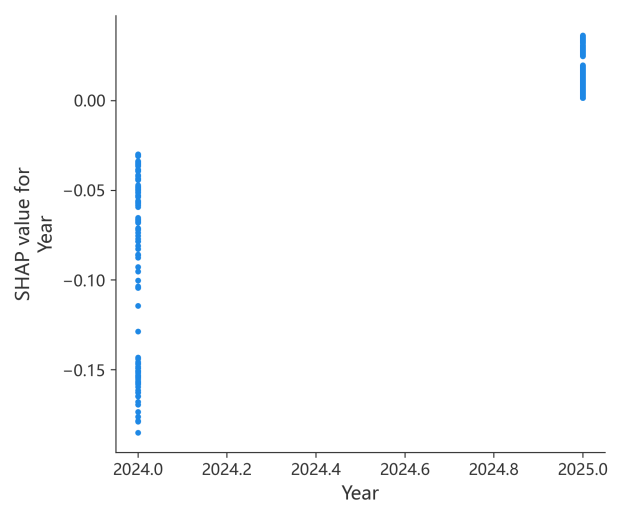 | 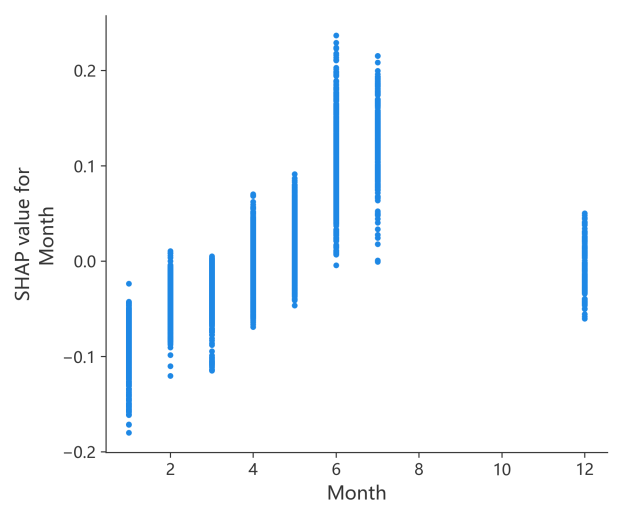 |
| G | H |
| 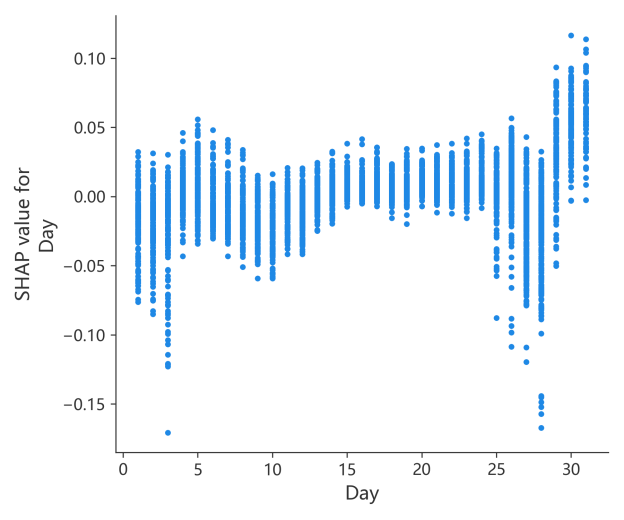 | 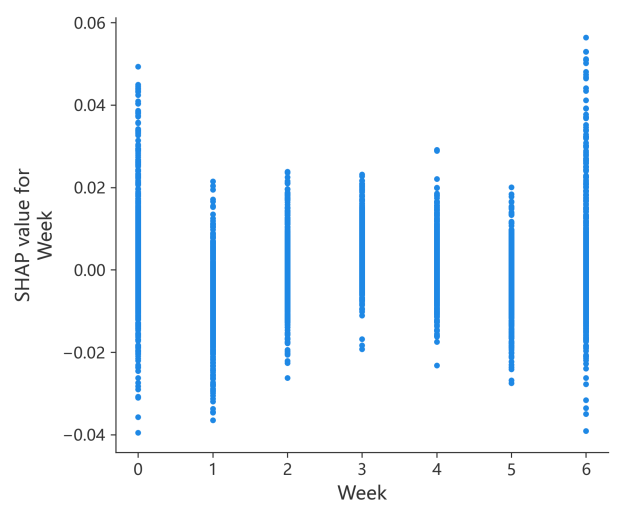 |
| I | J |
| 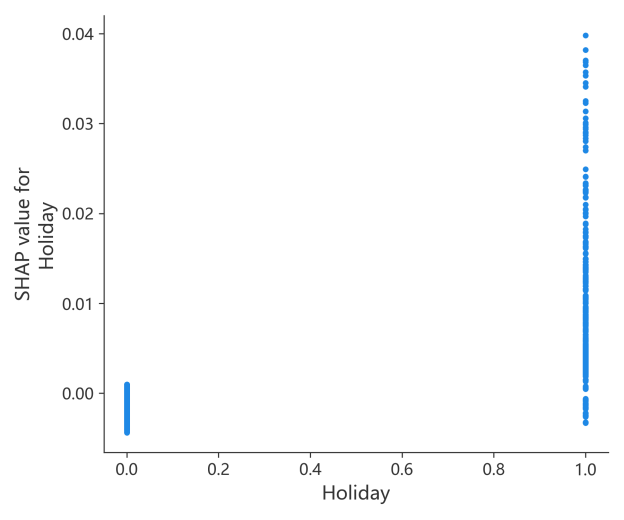 |  |
| K |  |
